# Precision modification of the human gut microbiota targeting surface-associated proteins

**DOI:** 10.1038/s41598-020-80187-3

**Published:** 2021-01-14

**Authors:** Raquel Marcos-Fernández, Lorena Ruiz, Aitor Blanco-Míguez, Abelardo Margolles, Borja Sánchez

**Affiliations:** 1 Department of Microbiology and Biochemistry of Dairy Products, Instituto de Productos Lácteos de Asturias-Consejo Superior de Investigaciones Científicas (IPLA-CSIC), Paseo Río Linares s/n, 33300 Villaviciosa, Asturias Spain; 2 Functionality and Ecology of Beneficial Microbes (MicroHealth) Group, Instituto de Investigación Sanitaria del Principado de Asturias (ISPA), Oviedo, Asturias Spain

**Keywords:** Biotechnology, Applied microbiology, Microbiology, Microbial communities, Microbiome

## Abstract

This work describes a new procedure that allows the targeted modification of the human gut microbiota by using antibodies raised against bacterial surface-associated proteins specific to the microorganism of interest. To this end, a polyclonal antibody recognising the surface-associated protein Surface Layer Protein A of *Lactobacillus acidophilus* DSM20079^T^ was developed. By conjugating this antibody with fluorescent probes and magnetic particles, we were able to specifically identify this bacterium both in a synthetic, and in real gut microbiotas by means of a flow cytometry approach. Further, we demonstrated the applicability of this antibody to deplete complex human gut microbiotas from *L. acidophilus* in a single step. *L. acidophilus* was found to interact with other bacteria both in synthetic and in real microbiotas, as reflected by its concomitant depletion together with other species. Further optimization of the procedure including a trypsin step enabled to achieve the selective and complete isolation of this species. Depleting a single species from a gut microbiota, using antibodies recognizing specific cell surface elements of the target organism, will open up novel ways to tackle research on the specific immunomodulatory and metabolic contributions of a bacterium of interest in the context of a complex human gut microbiota, including the investigation into therapeutic applications by adding/depleting a key bacterium. This represents the first work in which an antibody/flow-cytometry based application enabled the targeted edition of human gut microbiotas, and represents the basis for the design of precision microbiome-based therapies.

## Introduction

During the last 10 years there have been incredible advances in our understanding of how the human gut microbiota contributes to both health and disease. The human microbiota, especially the gut microbiota, has been considered an “essential organ” as these microbial communities that colonize our body provide us with essential metabolic, immune and physiological functions through their collective genome, which comprises approximately 150 times more genes than our own genome^1^. Relative stability of the intestinal microbiota configuration is achieved with the introduction of solid foods and the establishment of a relatively complex community dominated by obligate anaerobes^2,3^. After birth about 100 bacterial species colonize the intestine, increasing up to 10^3^ bacterial species in adulthood, with constantly evolving composition^2,4–6^. The main phyla that configure the intestinal microbiota are *Firmicutes*, *Bacteriodetes and Actinobacteria*, but many others, such as *Proteobacteria*, *Fusobacteria*, *Verrucomicrobia*, *Tenericutes* and *Lentisphaerae,* are present. The most abundant genera are *Bacteroides*, *Clostridium*, *Faecalibacterium*, *Eubacterium*, *Ruminococcus*, *Peptococcus*, *Peptostreptococcus*, *Lactobacillus*, *Streptococcus*, *Streptomyces* and *Bifidobacterium*^7,8^. The specific gut microbiota configuration is determined by several internal and external factors including age, race, diet, maternal colonization, as well as exposures to xenobiotics and antibiotics^9–12^. In addition, other factors such as stress, travel or drug treatment leads to quick changes in its composition^7^.

The complexity of the gut microbiota hampers to deduce the individual contribution of a species/strain to a given phenotype, condition or disease^13^. For this reason, the development of methods enabling the targeted modification of a microbiota, particularly focused on depleting a specific microbial group from a complex bacterial community, could significantly aid to decipher how a single species within a complex microbial community contributes to a disease or to a physiological, immune or metabolic process. In this study, we modified a synthetic microbiota using an antibody directed to the surface layer protein of *Lactobacillus acidophilus*. S-layer or surface layer is part of the cell envelope in some bacteria and archaea^14^. Several *Lactobacillus* species possess an S-layer such as *L. acidophilus, L. brevis, L. crispatus, L. buchneri, L. amylovorus, L. gallinarum, L. helveticus, L. johnsonii, L. fermentum* and *L. gasseri*^15^. The mature S-layer proteins from lactobacilli have a molecular mass in the range of 25–71 kDa^16^. The S-layer proteins of *L. acidophilus* have been associated with diverse roles in cell division, intestinal adhesion, and host immunomodulation^17^, and are known to protect cells against hostile environmental agents. Although *L. acidophilus* S-layer is composed predominantly by the S-layer protein SlpA, there are less-represented S-layer proteins also displayed on the bacterial surface, such as the S-layer protein SlpX. In this regard, an increase of *slpA* and *slpX* expression under salt stress was detected in *L. acidophilus* ATCC 4356^18^. Also, deletion of *slpX* in *L. acidophilus* ATCC 4356 exhibited lower growth rates, increased sensitivity to sodium dodecyl sulfate, and greater resistance to bile^19^. The sequences of the protein domains corresponding to the cell wall binding and self-assembly modules are variable among the S-layer proteins from lactobacilli^20^*.* For example, the C-terminal region of the SlpA protein from *L. acidophilus* ATCC 4356 was shown to be responsible for the cell wall binding and the N-terminal region for self-assembly^15^.

Developed in the late 1960s, flow cytometry (FC) is predominantly used in the biomedical field for the analysis of mammalian cell phenotypes using fluorescence-conjugated antibodies that recognize specific surface-associated proteins. On the contrary, use of FC in bacteriology has been limited due to the smaller size of bacteria and the lack of specific markers. Instead, non-specific fluorescent dyes targeting microbial macromolecules, such as DNA or proteins, IgA or IgG opsonized bacteria or the use of plasmid encoded fluorescent proteins are often used with bacteria^21^. There are many more examples in the literature showing that FC is an effective platform for single cell analysis on microbial cells^22,23^. Further applications based on the use of specific antibodies and FC have also been reported. For instance, recently, a method using reverse genetics allowed targeted isolation and culture of yet-uncultured microorganisms using a similar, targeted antibody generation^24^.

In this context, our group has recently reported the detection of *Lactobacillus rhamnosus* using a polyclonal and a monoclonal antibody directed to its cell wall hydrolase by using FC^25^. Herein, we propose a further step forward demonstrating the applicability of antibodies targeting specific surface-associated proteins to achieve precise human gut microbiota modification. In particular, this work reports on the targeted modification of gut microbiota communities through the use of an antibody raised specifically against the Surface-Layer Protein A (anti-SlpA) from *Lactobacillus acidophilus* DSM20079^T^, and involves the use of FC to monitor the progress of the successful depletion/enrichment of the target strain.

## Results

### Detection of *L. acidophilus* using a polyclonal anti-SlpA antibody

For enrichment and depletion of *L. acidophilus* from a synthetic or real microbiota, we developed a protocol based on using antibodies and magnetic beads. The polyclonal anti-SlpA antibody was fluorescently labelled with fluorescein isothiocyanate (FITC) or allophycocyanin (APC). The availability of an anti-APC antibody conjugated to magnetic beads allows the targeted isolation of those *L. acidophilus* cells that have been bound to the antibodies (see Material and Methods for details).

**Figure 1:**
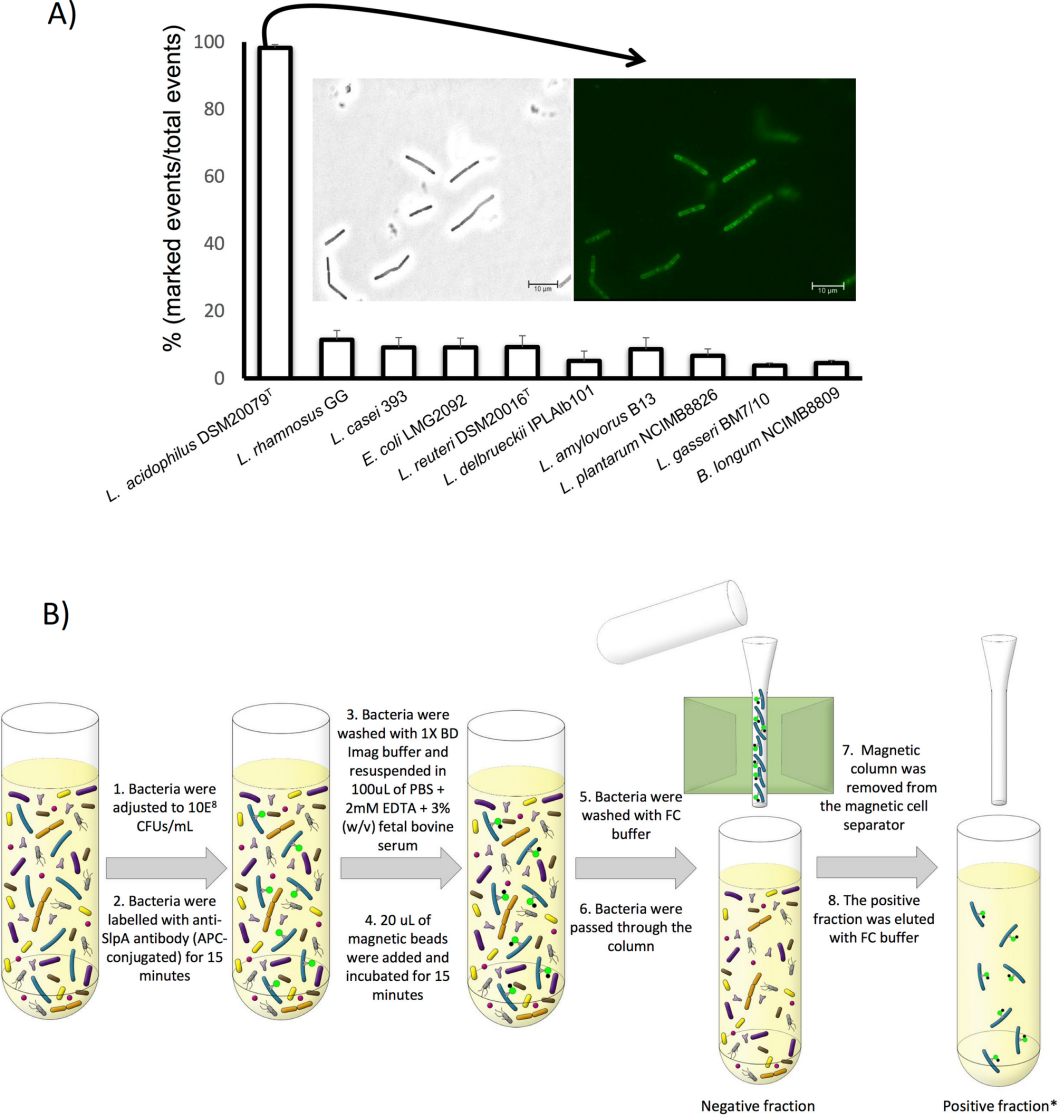
**(A)** Bar charts showing the percentage of fluorescent bacteria (y axis) labelled with the anti-SlpA polyclonal antibody conjugated to FITC in different lactobacilli and in *Bifidobacterium longum* and *E. coli* (x axis). Data are represented as the mean ± SD of at least six independent experiments. A phase contrast photography (left picture) and immunofluorescence microscopy photography (right picture) showing the binding of the FITC-anti SlpA antibody to the surface of *L. acidophilus* DSM20079^T^ is also shown. **(B)** Schematic diagram of the protocol for enrichment and depletion of *L. acidophilus* from a synthetic microbiota. * Incubation after step 5 is required in order to eliminate *L. acidophilus* associated bacteria.

Eight *Lactobacillus* strains, *Lactobacillus casei* ssp. *rhamnosus* GG, *Lactobacillus acidophilus* DSM20079^T^, *Lactobacillus amylovorus* B13, *Lactobacillus casei* 393, *Lactobacillus delbrueckii* ssp. *delbrueckii* IPLAlb101, *Lactobacillus reuteri* DSM20016^T^, *Lactobacillus gasseri* BM7/10, and *Lactobacillus plantarum* NCIMB 8826, were chosen as being representative of the *Lactobacillus* genus. *Bifidobacterium longum* subsp. *longum* and *Escherichia coli* were also considered as outgroups representing other bacterial phyla commonly present in the human gut microbiota. The labelling of the ten bacterial species with the anti-SlpA polyclonal antibody was evaluated by FC and analysis of dispersion diagrams. These were obtained by separating the bacteria in FC according to Side Scatter (SSC), Forward Scatter (FSC) and their fluorescence in the FITC channel, designating three regions in the plots corresponding to P1 (bacterial cells); LR2 (unlabelled bacteria) and UR2 (fluorescent bacteria). The anti-SlpA demonstrated a very specific recognition of *L. acidophilus* DSM20079^T^ cells, with virtually 100% of bacteria labelled after 15 min of incubation (Fig. 1A). The rest of the bacteria showed labelling percentages lower than 15% as deduced by FC, and showed no fluorescence at the confocal microscope (Supplementary Fig. 1).

### Specificity of polyclonal antibody against *L. acidophilus* surface layer protein A

**Figure 2:**
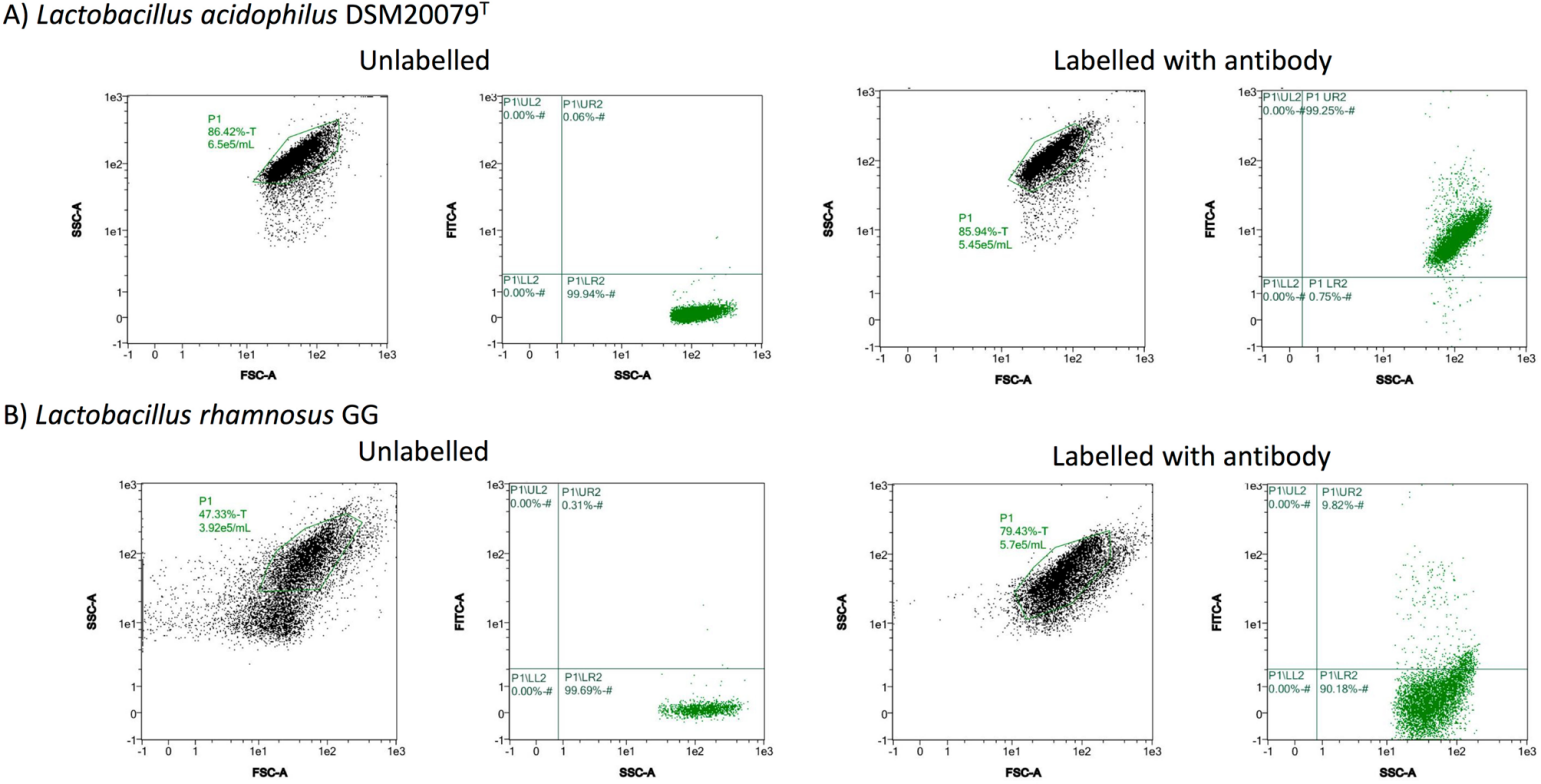
Dispersion diagrams of representative FC experiments showing the acquisition of labelled and non-labelled *L. acidophilus* DSM20079^T^ **(A)** and *L. rhamnosus* GG **(B)** cell suspensions in exponential phase of growth.

As polyclonal anti-SlpA antibody bound to *L. acidophilus* cells more efficiently than to the rest of species tested, even than to those containing S-layer proteins such as *L. gasseri*, we performed further analyses to determine which part of the cells was selectively recognized by the antibody and to confirm whether the antibody could recognize any structures in other bacterial species. Immunofluorescence microscopy of the ten bacterial species previously stained with FITC-anti SlpA antibody, showed that antibody binding was located on the surface of *L. acidophilus* cells, whereas fluorescence was undetectable in the rest of species assayed in this work. This contrasted with the 10–15% of fluorescent cells detected in the FC experiments for those species assayed, events that even while considered positive, presented fluorescence emission values drastically lower than *L. acidophilus* (Fig. 1A). The higher emission of *L. acidophilus* compared to the lower emissions of the rest of bacteria can be easily deduced comparing the positive events displayed in the dispersion plots of *L. acidophilus* DSM20079^T^ (98.29 ± 0.99%, Fig. 2A) to those of *L. rhamnosus* GG (11.47 ± 2.74%, Fig. 2B). The dispersion plots for other tested bacteria are included in Supplementary Fig. 1. The response of the bacteria to the pre-immune serum was also evaluated by FC, returning values lower than 8% in all bacteria (Supplementary Fig. 2). The specificity of the polyclonal antibody was also evaluated through Western blotting, using the extracellular protein fractions of the eight bacteria included in the synthetic microbiota, showing a sharp blot of the right molecular weight with the *L. acidophilus* fraction, but not with the other bacterial fractions (Supplementary Fig. 3).

**Figure 3:**
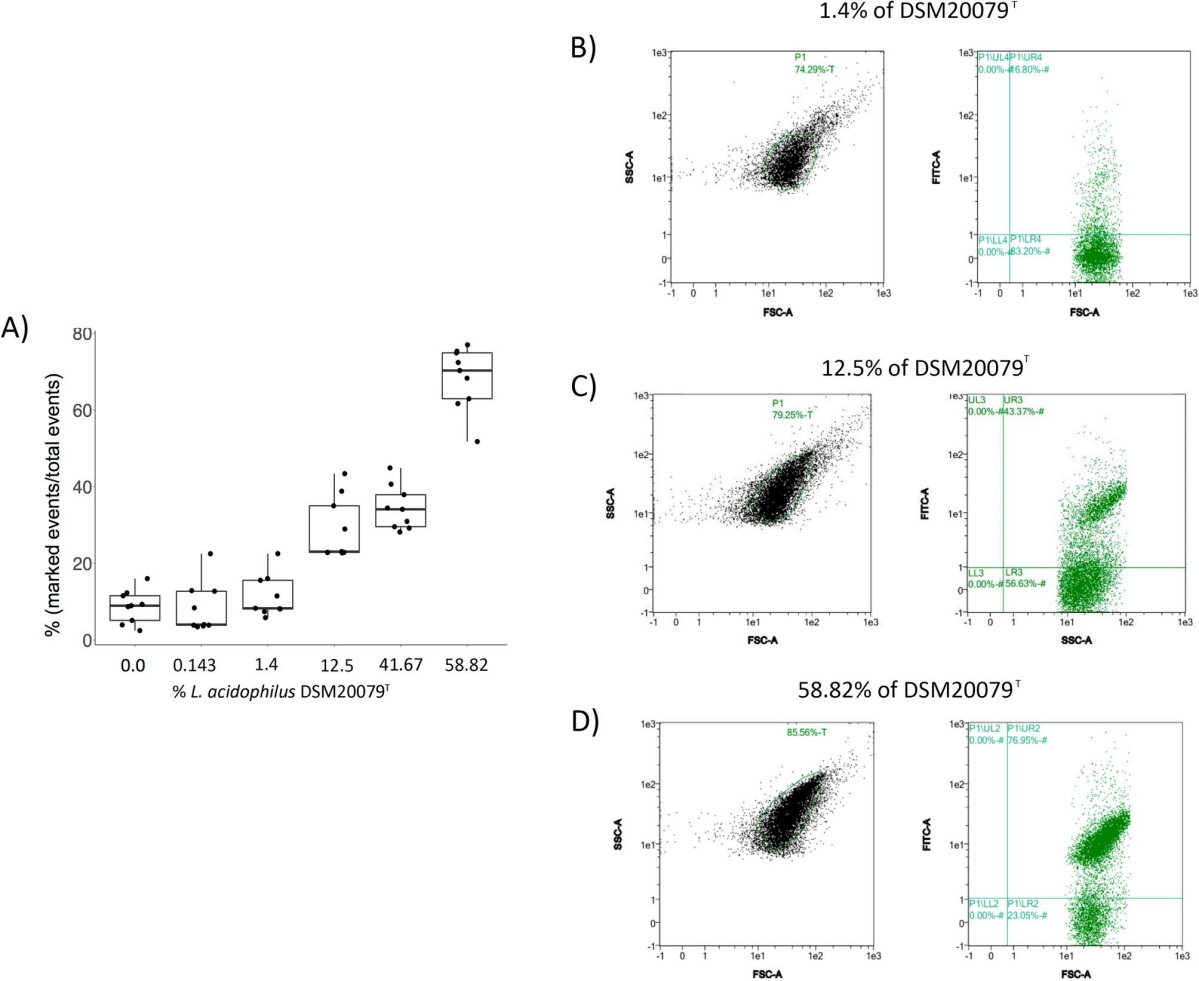
**(A)** Boxplot/jitter diagrams showing the percentage of *L. acidophilus* DSM20079^T^ cells labelled in a defined bacteria consortium representing a synthetic human gut microbiota. Data are represented as the mean ± SD of at least three independent experiments. As described in the M&M section, all bacteria were added in the same volumetric proportion with the exception of *L. acidophilus*, which was either absent or added at different percentages representing different proportions: 0.143%, 1.4%, 12.5%, 41.67% and 58.82%. (0.01:1, 0.1:1, 1:1, 5:1 or 10:1 ratios in term of volumes). The detection of DMS20079^T^ in a defined bacteria consortium was assessed in triplicate. **(B–D)** Dispersion diagrams of a representative FC experiment showing the detection of *L. acidophilus* DSM20079^T^ in a defined bacteria consortium when added at 1.4 **(B)**, 12.5 **(C)**, and 58.82 **(D)** percentages (volumetric proportion DSM20079^T^: rest of bacteria, 0.1:1, 1:1, and 10:1, respectively).

### Detection of *L. acidophilus* in a synthetic gut microbiota using flow cytometry

Given the high specificity of the anti-SlpA antibodies, we tried to specifically label the *L. acidophilus* DSM20079^T^ strain when included in a defined bacterial consortium containing representatives of the major human intestinal bacterial Phyla: *B. longum* subsp. *longum* NCIMB8809 (Actinobacteria), *E. coli* LMG2092 (Proteobacteria), *Blautia obeum* ATCC 29174, *Faecalibacterium prausnitzii* M21 and *L. gasseri* BM7/10 (Firmicutes), *Bacteroides thetaiotaomicron* VPI-5482 (Bacteroidetes) and *Akkermansia muciniphila* ATCC BAA-835 (Verrucomicrobia), all added in equal volumes using OD_600_ ≈ 0.2 cell suspensions. *L. acidophilus* DSM20079^T^ cells were added to this mix at different percentages: 0.143%, 1.4%, 12.5%, 41.67% and 58.82% (0.01:1, 0.1:1, 1:1, 5:1 or 10:1 vol/vol), being detectable even in the lower percentage value. When all bacteria were added at 1:1 proportion, and therefore *L. acidophilus* represented around 12.5% of bacteria present in the mix, *L. acidophilus* population was evident in the dispersion plots and, in those mixes including percentages of *L. acidophilus* from 12.5% to 58.82%, the proportion of events displaying fluorescence in the FITC channel increased with the *L. acidophilus* representation in the mix (Fig. 3A). The dispersion diagrams of the bacterial community mixes tested are included in Fig. 3B–D and Supplementary Fig. 4.

**Figure 4:**
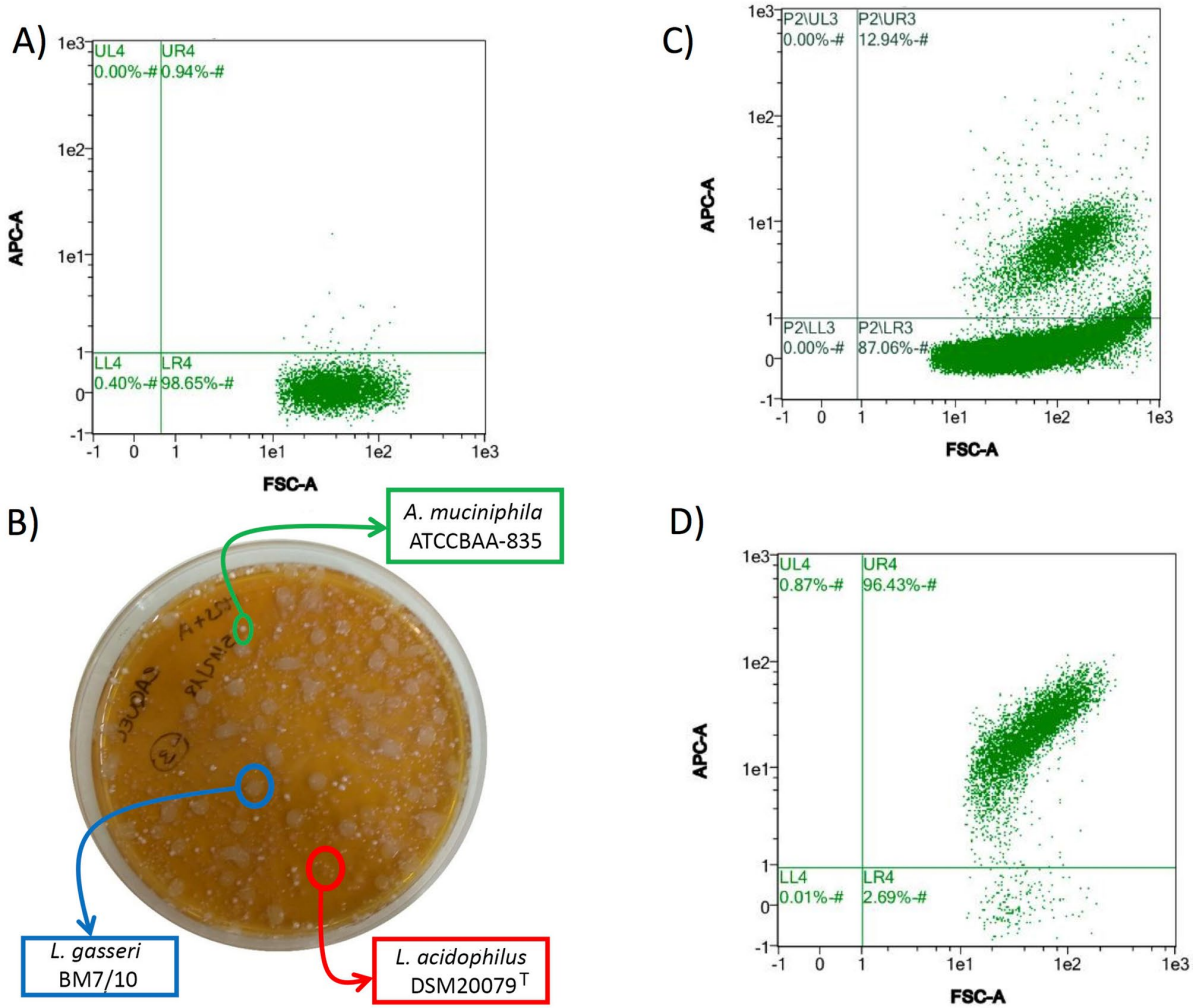
**(A)** Dispersion diagrams of representative FC experiments showing the APC-specific fluorescence in a synthetic microbiota depleted of *L. acidophilus* DSM20079^T^. **(B)** Representative Petri dish showing the bacteria corresponding to *L. acidophilus* DSM20079^T^ enriched fraction and their identifications based on 16S rRNA gene sequencing is also represented. **(C)** Dispersion diagrams of representative FC experiments in the *L. acidophilus* DSM20079^T^ enriched fraction showing the presence of non-fluorescent bacteria, and **(D)** in the *L. acidophilus* DSM20079^T^ enriched fraction with a previous treatment with trypsin, and which contained only fluorescent bacteria corresponding to *L. acidophilus*.

### Enrichment and depletion of *L. acidophilus* from a synthetic microbiota

In the next step, we combined the use of the anti-SlpA IgG polyclonal antibody conjugated to APC, with an anti-APC antibody conjugated to magnetic beads to specifically enrich *L. acidophilus* in a synthetic microbiota including the bacterial strains previously listed as representatives of *B. longum, E. coli, Bl. obeum, F. praustnizii, L. gasseri, B. thetaiotaomicron, A. muciniphila* and *L. acidophilus*, each bacterium representing a 12.5% of the mix. Using this approach, we were able to deplete the synthetic microbiota from *L. acidophilus* DSM20079^T^ cells in a single step (Fig. 4A, left panel), although the positive fraction retained in the magnetic column contained *L. acidophilus* together with *L. gasseri* and *A. muciniphila* cells, as evidenced by culture-dependent techniques and further 16S rRNA gene-based identification (Fig. 4B). Cell counts evidenced that 8.5 ± 2.4 × 10^5^ *A. muciniphila* and 1.4 ± 1.2 × 10^6^ *L. gasseri* cells were recovered after positive *L. acidophilus* enrichment, which indeed represented two logs lower than the recovered *L. acidophilus* concentration (1.0 × 10^8^). The presence of these bacteria in the positive fraction was evident during FC analysis as they appeared as events that did not emit fluorescence when analysed in the APC emission channel (Fig. 4C). In a previous work, we showed how bacterial clumps characteristic of stationary-phase cultures of *L. rhamnosus* GG were efficiently disaggregated by trypsin ^26^. In the same way, herein the inclusion of a single 15 min trypsin treatment prior to the magnetic selection protocol, resulted in the selective and exclusive isolation of *L. acidophilus* cells in a single step, as revealed by a very limited presence of non-fluorescence events in the positive fraction, and confirmed by culture-dependent and 16S rRNA analyses (Fig. 4D).

### Detection, enrichment and depletion of *L. acidophilus* in a real gut microbiota

**Figure 5:**
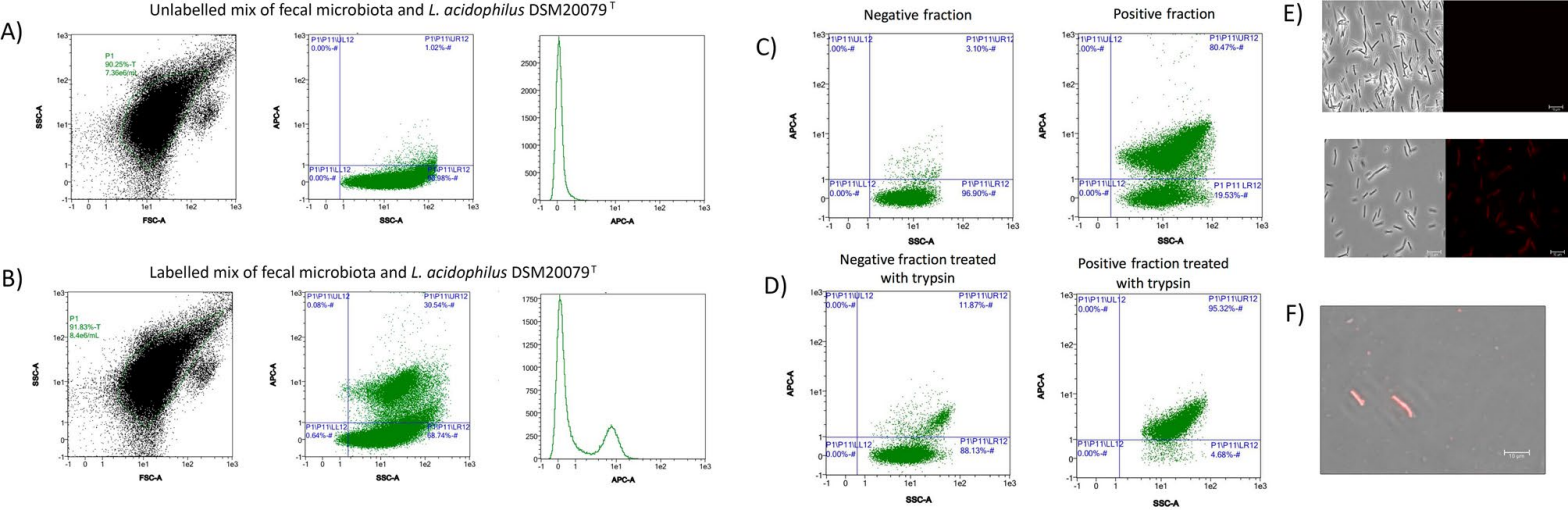
Dispersion diagrams of representative FC experiments showing the acquisition of unlabelled **(A)** and labelled **(B)** fecal gut microbiota supplemented with *L. acidophilus* DSM20079^T^. **(C)** Positive and negative fractions of a microbiota supplemented with DSM20079^T^ without trypsin pre-treatment. **(D)** Positive and negative fractions of a microbiota supplemented with DSM20079^T^ with trypsin pre-treatment. **(E)** Immunofluorescence microscopy photography showing *L. acidophilus* labelled and non-labelled with polyclonal antibody anti-SlpA conjugated with APC. **(F)** Immunofluorescence microscopy photography showing the binding of the APC-anti SlpA antibody to the surface of positive fraction after trypsin treatment.

As a proof-of-principle validation, the application of the developed antibody and above described procedure to detect, enrich and deplete *L. acidophilus* on synthetic microbiotas was validated on real human gut microbiota samples. For this purpose, five samples of real human gut microbiotas obtained from healthy adult donors were supplemented in an approximate proportion of one *L. acidophilus* per ten microbial cells, as explained in the Material and Methods section. By using this approach, the population of *L. acidophilus* cells were perfectly discriminated from the rest (and non-fluorescent) microbiota cells (Fig. 5; Supplementary Fig. 5).

Enrichment and depletion of *L. acidophilus* from the fecal microbiotas (n = 5) also followed the same trend as the synthetic microbiota (Fig. 5; Supplementary Fig. 5). Fecal microbiotas were depleted from *L. acidophilus* in a single step (Fig. 5C and Supplementary Fig. 6), although plating of these positive fractions evidenced the co-isolation (around 2 logs less in terms of colony numbers) of different isolates showing 16S rDNA sequence homology to *Streptococcus salivarius* (92%), *St. gallolyticus* (91%) and *Tumebacillus flagelatus* (94%) (data not shown). In order to disaggregate the *L. acidophilus* DSM20079^T^ cells from the rest of associated bacteria, a single 15-min trypsin pre-treatment was applied prior to the magnetic separation as previously described. With this technique, positive fractions contained around 95% of *L. acidophilus* cells as determined by FC analysis. However, a minimal number of fluorescent events were also retrieved in the negative fraction (Fig. 5D). The positive and negative controls were *L. acidophilus* DSM20079^T^ labelled and unlabeled (Fig. 5E). As additional validation, confocal microscopy corroborated that trypsin treatment did not disrupt the antibody interaction (Fig. 5F).

**Figure 6:**
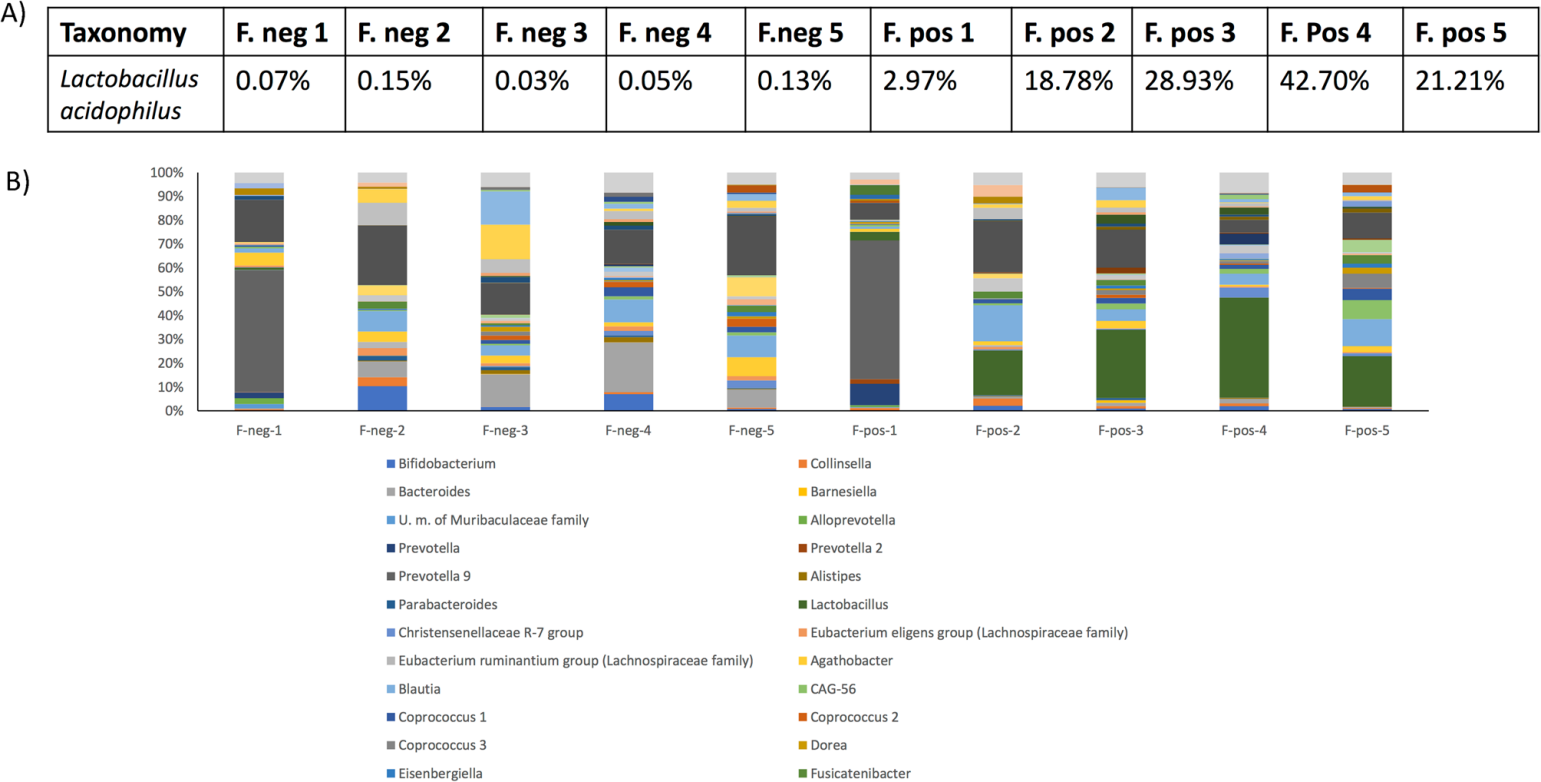
**(A)** Relative abundance of *Lactobacillus* in depleted (negative) and enriched (positive) fractions of five microbiotas supplemented with *L. acidophilus.* **(B)** Genera present in positive and negative fractions of the microbiotas supplemented with *L. acidophilus.*

### Microbial profile of positive and negative fractions

To further check the relative abundance of *L. acidophilus* in the negative (or depleted) and positive (or enriched) fractions of gut microbiota, a 16S rRNA gene sequencing analysis was performed on fractions obtained following the procedure described above, on five different microbiotas supplemented with *L. acidophilus* DSM20079^T^. The results show that, in all the microbiotas analysed, the genus *Lactobacillus* was significantly enriched in the positive fraction, and depleted in the negative fraction, where it represented values ranging from 0.02 to 0.15%. The enrichment of *Lactobacillus* in the positive fractions varied between 40 and 1,000-fold as compared to the corresponding negative fractions (Fig. 6). Within the *Lactobacillus* sequences, the large majority of them were identified as belonging to the species *L. acidophilus* (data not shown).

## Discussion

The identification of specific surface-associated proteins and development of antibodies specifically recognizing them, may contribute to the conception of FC approaches in the field of microbiology. As a proof of concept, we applied this methodology to the *L. acidophilus* species, showing that is not only possible to detect a specific species in a complex microbiota, but also to perform a targeted depletion, and even a further enrichment, in a single step.

To this end, we have analyzed how a polyclonal antibody raised against the surface-associated S-layer protein A of *L. acidophilus* can be applied in its detection through a FC approach. Besides, confocal laser microscopy analysis evidenced that, from the bacterial species tested in this study, only *L. acidophilus* cells were efficiently labelled with this serum. Remarkably, some positive events were identified through FC for those strains that evidenced no labelling through immunofluorescence microscopy. This is important as the polyclonal antibody presented in this work may contain other antibodies recognizing other bacterial targets that are usually present in the surface of the gut bacteria. The higher sensitivity of FC with respect to the fluorescence microscopy represents one of its main advantages, and generation of a monoclonal antibody would decrease the false positive rates obtained with our polyclonal anti-SlpA antibody^27^.

The polyclonal antibody against the surface associated S-layer protein A also allowed for the detection of *L. acidophilus* cells when present in a synthetic microbiota comprised of 8 representative gut bacterial species. Furthermore, the same antibody enabled the specific depletion of this bacterium from the community in a single step. Applying the same methodology over fecal microbiotas, we showed that it is possible to deplete a complex gut microbial population from *L. acidophilus* cells in a single step and, that by using a pre-treatment with trypsin, we could prevent the concomitant isolation of other species likely associated with the target microorganism within the community. These findings are very relevant as, for the first time, a procedure for obtaining a kind of “Knock-out” microbiota depleted of one specific bacterial species, is described. This may represent a novel methodology that will aid to understand the specific contribution of a single bacterium to a given phenotype or effect associated with a microbial community. However, such approaches would benefit from the development of more specific and monoclonal antibodies.

Many diseases are associated with alterations in the gut microbiome, including diabetes, obesity, inflammatory bowel disease, atherosclerosis, alcoholic liver disease, non-alcoholic fatty liver disease, cirrhosis cancer or metabolic syndrome^28–30^. Changes in the diet, use of probiotics, prebiotics or antibiotics are common means to achieve microbiota modifications, although they are not very selective, at least at species level^31^. In a recent work, tungstate was used to selectively limit the expansion of the members of the *Enterobacteriaceae* family, which is characteristic from active inflammatory bowel disease^32^. Also, the work of Hsu and colleagues provides an insight into the ecological importance of phages as modulators of bacterial colonization, and it additionally suggests the potential impact of gut phages on the mammalian host with implications for their therapeutic use to precisely modulate the microbiome^33^. The technique presented in this work represents a novel methodology for the precise modification of a microbiota, in this case targeting the *L. acidophilus* species. Our results showed that it is possible to deplete a microbiota from *L. acidophilus* cells in a single step using a pre-treatment with trypsin. This method could be applied to the majority of *L. acidophilus* strains due to the high similarity and conservation of the SplA between the strains of this species (Supplementary Fig. 7). Other *Lactobacillus* strains possess an S-layer such a*, L. crispatus, L. helveticus* or and *L. gasseri,* and these proteins are also present on the surface of other Bacteria and Archaea representatives^34^. The results of the 16S rRNA gene sequencing analysis show that *L. acidophilus* is enriched in the positive fraction, whereas the percentage of *L. acidophilus* that remains in the negative fraction is minimal. The described procedure could likely be further optimized to achieve a percentage of *L. acidophilus* in the negative fraction closer to 0% by increasing the anti-APC used, since the concentration of antibody and beads influences the depletion capacity.

Theoretically, any bacteria presenting specific targets on their surface, could be precisely depleted from a gut microbiota following the protocol described in this paper, by employing antibodies raised specifically against selected particular protein markers from the target bacterial species. For any bacteria of interest, the application of a methodology like the one described in this work would firstly need to detect major proteins present on the bacterial surface by using, for instance, SDS-PAGE or surface shaving methodologies^35^. The procedure herein described is clearly a different and more specific technique than other procedures for targeted microbiota modification described to date, such as metagenomic engineering through conjugative transfer^36^. The implications of this technique in microbiome-based therapies may be of great importance. For instance, the absence of *A. muciniphila* has been associated with resistance to PD-1 chemotherapy in melanoma^37,38^, lower abundances of *F. prausnitzii* are associated with Inflammatory Bowel Disease, and presence of specific bacteria such as *E. coli* Pks^+^, *Bacteroides fragilis* Btf + and *Fusobacterium nucleatum* is related to colo-rectal cancer^39,40^. Targeted modification of a gut microbiota will enable depletion and/or enrichment of native microbiotas with these bacteria, for instance eliminating specific *E. coli* strains associated with certain conditions, or enriching commensal beneficial bacteria such as *F. prausnitzii* on individual fecal microbiotas, with a clear potential application in gut microbiota auto-transplant.

To date, most studies use probiotics or prebiotics to restore the composition of the gut microbial community and modify the functions of the microbiome and the phenotype of the host. Such approaches, however, do not allow to study the biological effect derived from the removal of a particular bacterium from the microbiota^41^. The methodology described in this work represents a novel technique providing proof of concept for the detection, enrichment and depletion of specific bacteria in a bacterial consortium, or in a more complex microbiota, without the requirement of possessing a FACS (Fluorescence-Activated Cell Sorting) sorter in the research facilities. By facilitating precision modification of a microbiota, this new approach will be of great relevance to those research groups interested in the study of the contribution of single species to different diseases, physiological, metabolic or immune processes, including fecal microbiota transplantation. We are currently using our methodology to deplete or eradicate intestinal microbiota from strains/species and applying this model to the study of how they contribute to a certain disease.

## Material and methods

### Ethics statement

Ethics approval for this study (reference code AGL2016-78311-R) was obtained from the Bioethics Committee of CSIC (Consejo Superior de Investigaciones Científicas) and from the Regional Ethics Committee for Clinical Research (Servicio de Salud del Principado de Asturias; no. 127/16) in compliance with the Declaration of Helsinki. All determinations were performed with fully informed written consent from all participants involved in the study.

### Fecal sample collection and microbiota extraction

The study sample comprised 5 fecal samples from healthy donors. No donors reported any severe diseases in the last 6 months. Fresh fecal material was collected in a sterile container and immediately manipulated and homogenized within a maximum time span of 2 h. Nine millilitres of sterile NaCl 0.9% (w/v) was added to 1 g of sample, and the mixture was homogenized in a sterile bag, using a laboratory paddle blender (Stomacher Lab Blender 400, Seward Ltd. UK) for 1 min. Microbiota extraction was then performed following the protocol described by Hevia and coworkers^42^. A solution of Nycodenz 80% (w/v) (Progen Biotechnik GmbH, Heidelberg, Denmark) was prepared in ultrapure water, and sterilized at 121 °C for 15 min. A volume of 3 mL of the diluted, homogenized fecal sample was placed on top of 1 mL of the Nycodenz solution, and centrifuged for 40 min at 4 °C (10,000×*g*, MLS-50 Swinging-Bucket Rotor, Beckman Coulter, Indianapolis, IN). The upper phase (soluble debris) was discarded after centrifugation, and the layer corresponding to the microbiota was collected, washed once and resuspended in 1 mL of FC buffer (1 × MACSQuant Running Buffer, MILTENYI BIOTEC, Germany).

### Bacterial strains and growth conditions

*L. casei* ssp. *rhamnosus* GG (ATCC53103), *L. acidophilus* DSM20079^T^, *L. amylovorus* B13, *L. casei* 393, *L. delbrueckii* ssp. *delbrueckii* IPLAlb101, *L. reuteri* DSM20016^T^, *L. gasseri* BM7/10 and *L. plantarum* NCIMB 8826 were grown in MRS (Biokar Diagnostics, France). *B. longum* subsp. *longum* NCIMB8809 was grown in MRS supplemented with 0.05% (w/v) L-cysteine (MRSC, SIGMA, St. Louis, MO). *E. coli* LMG2092 was grown in Luria–Bertani broth (LB) containing 10 g/L tryptone (Biokar Diagnostics), 5 g/L yeast extract (BIOKAR DIAGNOSTICS), and 10 g/L of NaCl (Merck, KGaA, Darmstadt, Germany). *Bl. obeum* ATCC 29174 and *B. thetaiotaomicron* VPI-5482 were cultivated in a combination of Reinforced Clostridial Broth (Oxoid, Ltd., Basingstoke, Hampshire, UK) and Brain–Heart Infusion (BHI, Oxoid) supplemented with 5% (v/v) heat-inactivated fetal bovine serum (Labclinics, Barcelona, Spain). *A. muciniphila* ATCC BAA-835 was grown in Anaerobe Basal Broth (Oxoid). *F. prausnitzii* M21 was grown in BHI supplemented with 0.5% w/v yeast extract, 1 g/L cellobiose (Panreac Applichem, Darmstadt, Germany), 1 g/L maltose (Panreac Applichem) and 0.5 g/L cysteine. All bacteria were first grown on the surface of agar plates from − 80 ºC stocks, with the exception of *Bl. obeum*, *B. thetaiotaomicron*, *A. muciniphila* and *F. prausnitzii* which were grown directly in fresh liquid media at 37 ºC in a MG500 anaerobic chamber (Don Whitley Scientific, West Yorkshire 100, UK; atmosphere of 10% (v/v) H_2_, 10% CO_2_, and 80% N_2_) for 48 h. These pre-cultures were used as fresh inoculum for the preparation of active cultures. In the case of *E. coli*, a constant shaking of 200 rpm was applied to favour growth of the bacterium.

### Purification of the surface-layer protein A

Purification of the Surface-Layer Protein A was performed after separation in SDS-PAGE from the extracellular protein fraction of the *L. acidophilus* DSM20079^T^ strain ^43^. Fifty mL aliquots of fresh MRSC broth were inoculated (1% v/v) from an overnight culture. Cultures were allowed to enter the stationary phase of growth and cells were harvested by centrifugation (9300×*g*, 4 ºC, 10 min). Supernatants were then filtered (0.45 μm) and extracellular proteins were precipitated by adding a final concentration of 6% (w/v) trichloroacetic acid (TCA; SIGMA). Extracellular proteins were allowed to precipitate at 4 °C for 2 h. Proteins were recovered by centrifugation (9300×*g*, 4 °C, 10 min); pellets were washed twice with 2 mL of chilled acetone (SIGMA) and dried at room temperature. Proteins were resolubilized by ultrasonication (Ultrasonic bath; Deltasonic, Meaux, France) in 200 μL of 1 × Laemmli buffer for 10 min^44^. Bands corresponding to SlpA were excised from the gels, pooled and used for polyclonal antibody generation.

### Polyclonal antibody generation

A polyclonal serum against the purified *L. acidophilus* DSM20079^T^ SlpA was generated at the Central Facilities of the University of Oviedo (Spain) as already described^45^. A rabbit was immunized five times, with an interval of 15 days between immunizations, with around 500 µg of protein dissolved in 1 mL of PBS, and mixed with 1 mL of Freund's incomplete Adjuvant. The rabbit was finally sacrificed by intracardiac puncture and blood was let to coagulate at 37 ºC for 4 h and subsequent overnight incubation at 4 ºC. Serum was separated by centrifugation (30 min, 2000 × *g*), and used for purifying the IgG. Firstly, ammonium sulphate was added to a final concentration of 45% (w/v), and the mix was incubated overnight at 4 ºC. After centrifugation (1 h, 10,000 × *g*, 4 ºC), the pellet was resuspended in 30 mL of PBS. This was extensively dialyzed against PBS, and loaded in a ProteinA Sepharose 4 Fast Flow, previously equilibrated with 10 column volumes of PBS (50 mL). The column was washed with 6 column volumes of PBS, and five fractions of 5 mL were eluted with citric acid 100 mM pH 3.0. pH was corrected in each aliquot by adding 1 mL of 1 M Tris–HCl pH 9.0. Fractions were mixed, centrifuged in a Vivaspin 20 device (3000 × *g*, molecular weight cut-off of 10 kDa) and washed with 20 mL of PBS. Protein concentration was estimated by measuring the A_280_ of the sample, aliquoted and stored at − 80 ºC.

The response of the bacteria used in this work to the pre-immune serum was evaluated by direct labelling of the serum with FITC or APC. Labelled and unlabelled bacteria, bacterial mixes and microbiotas were acquired and analysed by MACSQuant flow cytometer (MILTENYI BIOTEC).

### Antibody conjugation

The Polyclonal anti-SlpA serum IgG fraction was conjugated with FITC or APC using specific conjugation kits (Abcam Cambridge, MA, USA) and following manufacturer’s instructions. FITC is a green fluorescent dye that has an excitation wavelength maximum of 495 nm and an emission wavelength maximum of 519 nm. APC is an intensely bright phycobiliprotein isolated from red algae that exhibits far-red fluorescence with high quantum yields, with an absorbance maximum at 650 nm and a fluorescence emission peak at 660 nm. For FITC and APC conjugation, the anti-SlpA IgG polyclonal antibody fraction was reconstituted in phosphate buffered saline (PBS). One hundred microliters of a 1.5 mg/mL antibody dilution were added to the reactive dye for each conjugation event. Ten µL of FITC-Modifier reagent or APC- Modifier reagent was added to the antibody before adding the FITC or APC. The antibody-dye mixtures were incubated in the dark at room temperature for 3 h. After incubation, 10 µL of FITC-Quencher or APC-Quencher reagent was added and mixed gently. The concentration of conjugated antibody in the final sample was 20 µg/mL.

### Flow cytometry analysis

Labelled cells/microbiotas with anti-SlpA polyclonal antibodies were acquired and analysed in a MACS Quant Flow Cytometer device (MILTENYI BIOTEC) using the following acquisition parameters: flow rate set to “low”, uptake volume of 10 µL, FSC set to hyperlogarithmic amplification (438 V), SSC set to hyperlogarithmic amplification (535 V), channel B1 corresponding to the FITC detection set to hyperlogarithmic amplification (331 V) and channel R1 corresponding to the APC detection set to hyperlogarithmic amplification (360 V). At least 10,000 events were acquired in each run. At least eight independent experiments were conducted with the exception of the five intestinal microbiotas.

### Detection of individual bacteria

The binding specificity of the anti-SlpA antibody was tested over 10 different bacteria species, i.e. *L. rhamnosus* GG, *L. acidophilus* DSM20079^T^, *L. amylovorus* B13, *L. casei* 393, *L. delbrueckii* IPLAlb101, *L. reuteri* DSM20016^T^, *L. gasseri* BM7/10 and *L. plantarum* NCIMB8826, *B. longum* NCIMB8809 and *E. coli* LMG2092.

For each species, one millilitre of cultures in exponential phase of growth (OD_600_ ≈ 0.7) was centrifuged at 10,000×*g* for 5 min. After that, bacterial suspensions were adjusted to an OD_600_ = 0.2 (around 1E^8^ CFU/mL) using FC buffer. Twenty-five µL of bacterial suspensions were mixed with 25 µL of the FITC-conjugated antibody at a final concentration of 20 µg/mL. The samples were incubated for 15 min at room temperature and were then washed with bacterial FC buffer. Finally, bacteria were resuspended in 150 µL of FC buffer and aliquots of 10 µL were further analysed by FC.

### Detection of *L. acidophilus* in a gut microbiota

The detection of *L. acidophilus* in a mix of bacteria was investigated over a synthetic microbiota and over five samples of real gut microbiotas isolated from healthy donors.

A synthetic microbiota was configured using pure cultures of eight representative bacterial genera from the human gastrointestinal tract. This synthetic microbiota included *B. longum* subsp. *longum* NCIMB8809, *L. acidophilus* DSM20079^T^, *L. gasseri* BM7/10, *Bl. obeum* ATCC 29174, *B. thetaiotaomicron* VPI-5482, *A. muciniphila* ATCC BAA-835, *F. prausnitzii* M21 and *E. coli* LMG2092. Bacteria were grown individually in their respective media to an OD_600_ ≈ 0.7. After that, bacterial suspensions were adjusted to an OD_600_ ≈ 0.2 (10E^8^ CFU/ mL approximately) using FC buffer. All bacteria strains were added in the same proportions mixing equal volumes, but the proportions of *L. acidophilus* DSM20079^T^ were modulated in the mix by varying the final volume of the DSM20079^T^ cell suspension added (volume DSM20079^T^: volume rest of bacteria): 0% (0:1), 0.143% (0.01:1), 1.4% (0.1:1), 12.5% (1:1), 41.67% (5:1) and 58.82% (10:1). In the case of real gut microbiotas, *L acidophilus* DSM20079^T^ was grown until exponential phase OD_600_ ≈ 0.7 and adjusted to OD_600_ ≈ 0.2, and the extracted microbiota adjusted to an OD_600_ ≈ 5.0 representing approximately tenfold more microbial cells than the *L. acidophilus* suspension. *L. acidophilus* DSM20079^T^ was added to the adjusted microbiotas using a volumetric ratio of 1:1, which means that, approximately, 9.09% of the total microorganism counts theoretically corresponded to *L. acidophilus*.

Finally, both synthetic and real microbiotas were labelled with anti-SlpA polyclonal antibody conjugated to FITC, and data was acquired and analysed by MACS Quant flow cytometer, as indicated previously.

### Specific enrichment and depletion of *L. acidophilus* from a synthetic or a real microbiota

The enrichment and depletion of *L. acidophilus* from a mix of bacteria supplemented with *L. acidophilus* DSM20079^T^ cells in a volumetric 1:1 ratio (12.5% of each bacteria) configured with cell suspensions at an OD_600_ ≈ 0.2, or real gut microbiotas adjusted to OD_600_ ≈ 5.0, were tested. A schematic diagram of the enrichment and depletion method of *L. acidophilus* from a synthetic microbiota is represented in Fig. 1B. Mixes were labelled with the polyclonal anti-SlpA serum IgG fraction conjugated to APC and incubated for 15 min at room temperature. Then, mixtures were washed at 13,000 × g for 5 min and the supernatants removed. For positive selection of *L. acidophilus*, bacterial mixes were resuspended in 100 µL of resuspension buffer [RB: PBS supplemented with 2 mM EDTA and 3% (v/v) of de-complemented bovine foetal serum]. To this mix, 10 µL of magnetic anti-APC particles were added (BD BIOSCIENCES, San José, USA). The mixture was incubated for 15 min at 4 °C. Then, bacteria were washed at 13,000×*g* for 5 min with 1X BD IMag buffer (BD BIOSCIENCES). Magnetic MS columns were placed on a MiniMACS Separator (MILTENYI BIOTEC), and conditioned with 500 µL 1X BD IMag buffer (BD BIOSCIENCES). Bacteria were either resuspended in 500 µL RB or in 0.25% (w/v) Trypsin–EDTA solution (SIGMA). For the latter, mixes were incubated for 15 min at 37 ºC in order to eliminate all potential protein-mediated interactions among bacteria. Positive fraction was retained in the magnetic columns after three washes with 1X BD IMag buffer (BD BIOSCIENCES). Finally, the positive fraction was eluted by separation of the magnetic columns from the MiniMACS separator and after addition of 500 µL 1X BD IMag buffer. Both positive and negative fractions were analysed by FC and 16S RNA gene sequencing. Serial dilutions of the positive fractions were plated onto the surface of MRS agar plates, and the resulting colonies were identified by 16S rRNA gene amplification and sequencing (i.e. three colonies of intestinal synthetic microbiota and seven colonies of complex intestinal microbiota).

### Immunofluorescence microscopy

The binding specificity of the anti-SlpA antibodies contained in the polyclonal serum was also determined by confocal scanning laser microscope equipped with a Leica DFC365FX digital camera (DMi8, Leica Microsystems).

Firstly, the different bacterial species were grown individually until exponential phase OD_600_ ≈ 0.7, were then washed with FC buffer and incubated for 15 min with the polyclonal antibody anti-SlpA conjugated with FITC (200 µg/mL). In the case of positive fractions enriched in *L. acidophilus*, bacterial mixes were incubated for 15 min with the polyclonal antibody anti-SlpA conjugated to APC (200 µg/mL). Individual bacteria and positive fractions were resuspended in 10 µL of flow cytometer buffer and analysed with fluorescence microscopy using a 100 × oil objective. The images were acquired with software LasX (LEICA Microsystems). The FITC filter cube (excitation 480/40, emission 527/30) was used for labelled bacteria species, and for the positive fraction of the supplemented microbiota the APC filter cube (excitation 620/60, emission 700/75) was selected.

### Specificity of polyclonal antibody by Western Blot

Sodium dodecyl sulfate polyacrylamide gel electrophoresis (SDS-PAGE) and Western blot were performed as described below. First, proteins were separated using a polyacrylamide concentration of 14% (w/v) (Precast gels INVITROGEN, THERMO FISHER SCIENTIFIC, Waltham, MA) for 1 h at a constant intensity of 40 mA/gel. Proteins were visualized with Coomassie blue R-250 (THERMO FISHER SCIENTIFIC). Western blotting was performed with the objective of checking the signal of polyclonal antibody on the S layer protein A of *L. acidophilus* DSM20079^T^*.* Extracellular proteins were obtained following a previously described protocol^46^*.*

Proteins separated by SDS-PAGE were transferred and immobilized onto polyvinylidene fluoride (PVDF) membranes (GE HEALTHCARE, Madrid, Spain) for 30 min under a constant voltage of 30 V for one hour in a mini gel tank/blot module set (THERMO FISHER SCIENTIFIC). PVDF membranes were blocked with PBS (phosphate buffered saline, OXOID) supplemented with 0.1% (v/v) Tween-20 (PBST) (SIGMA) and with 5% (w/v) skimmed milk (PBST-L) (OXOID) for 3 h at RT under constant shacking. Membranes were washed twice and monoclonal/polyclonal antibodies were diluted to 1:2000 in PBST-L and incubated over the membranes (O/N, 4 °C) with slight agitation. Membranes were then washed 4 times with PBST and incubated for 1 h with a secondary antibody conjugated to horseradish peroxidase (horseradish peroxidase-conjugated anti-rabbit or anti-mouse IgG (SIGMA)), diluted to 1:1000 in PBST-L. For detection of immunoreactive bands, a commercial solution containing the chromogenic reagents chloronaphthol and diaminobenzidine (CN/DAB Substrate Kit, THERMO FISHER SCIENTIFIC) was used.

### 16S rRNA sequencing of positive and negative fractions

Partial 16S rRNA gene sequences were amplified from extracted DNA using primer pair Probio_Uni and/Probio_Rev, targeting the V3 region of the 16S rRNA gene sequence^47^. 16S rRNA gene amplification and amplicon checks were carried out as previously described^47^. 16S rRNA gene sequencing was performed using a MiSeq (ILLUMINA) at the DNA sequencing facility of GenProbio srl (https://www.genprobio.com) according to the protocol previously reported^47^. Following sequencing, the .fastq files were processed using a custom script based on the QIIME software suite^48^. Paired-end read pairs were assembled to reconstruct the complete Probio_Uni/Probio_Rev amplicons. Quality control retained sequences with a length between 140 and 400 bp and mean sequence quality score > 20 while sequences with homopolymers > 7 bp and mismatched primers were omitted. In order to calculate downstream diversity measures (alpha and beta diversity indices, Unifrac analysis), 16S rRNA Operational Taxonomic Units (OTUs) were defined at 100% sequence homology using DADA2^49^; OTUs not encompassing at least 2 sequences of the same sample were removed. Notably, this approach allows highly distinctive taxonomic classification at single nucleotide accuracy^49^. All reads were classified to the lowest possible taxonomic rank using QIIME2^48,50^ and a reference dataset from the SILVA database^51^.

The bacterial profile of each sample was represented through Bar plots. Only genera with relative abundance > 0.5% (or 1%) are shown. Moreover, the bacterial profile at species level has been predicted, which is to be considered as approximate. The 16S rRNA gene sequences have been deposited in the NCBI database with the Bioproject Accession number PRJNA655374 (biosamples from SAMN15733960 to SAMN15733969).

### Statistical analysis

Data were represented by mean ± SEM and differences between the bacterial labelled conditions were assessed at least in triplicate.

## Supplementary Information

Supplementary Figures.

## References

[1] Ursell LK (2014). The intestinal metabolome: An intersection between microbiota and host. Gastroenterology.

[2] Anwar, H. *et al.* Gut microbiome: A new organ system in body. in *Parasitology and Microbiology Research* (ed. Bastidas-Pacheco, G. A.) 1–20 (IntechOpen, 2020).

[3] Goedicke-Fritz S (2017). Preterm birth affects the risk of developing immune-mediated diseases. Front. Immunol..

[4] Davis MY, Zhang H, Brannan LE, Carman RJ, Boone JH (2016). Rapid change of fecal microbiome and disappearance of *Clostridium difficile* in a colonized infant after transition from breast milk to cow milk. Microbiome.

[5] Pelzer E, Gomez-Arango LF, Barrett HL, Nitert MD (2017). Review: Maternal health and the placental microbiome. Placenta.

[6] Kundu P, Blacher E, Elinav E, Pettersson S (2017). Our gut microbiome: The evolving inner self. Cell.

[7] Rea D (2018). Microbiota effects on cancer: From risks to therapies. Oncotarget.

[8] Fernández MF (2018). Breast cancer and its relationship with the microbiota. Int. J. Environ. Res. Public Health.

[9] Lu K, Mahbub R, Fox JG (2015). Xenobiotics: Interaction with the intestinal microflora. ILAR J..

[10] Nagpal R (2018). Gut microbiome and aging: Physiological and mechanistic insights. Nutr. Heal. Aging.

[11] Singh RK (2017). Influence of diet on the gut microbiome and implications for human health. J. Transl. Med..

[12] Francino MP (2016). Antibiotics and the human gut microbiome: Dysbioses and accumulation of resistances. Front. Microbiol..

[13] Langille MGI (2013). Predictive functional profiling of microbial communities using 16S rRNA marker gene sequences. Nat. Biotechnol..

[14] Sleytr UB, Schuster B, Egelseer E-M, Pum D (2014). S-layers: Principles and applications. FEMS Microbiol. Rev..

[15] Hynönen U, Palva A (2013). *Lactobacillus* surface layer proteins: Structure, function and applications. Appl. Microbiol. Biotechnol..

[16] Johnson B, Selle K, O’Flaherty S, Goh YJ, Klaenhammer T (2013). Identification of extracellular surface-layer associated proteins in *Lactobacillus acidophilus* NCFM. Microbiology.

[17] Johnson BR, Klaenhammer TR (2016). AcmB is an S-layer-associated β-N-acetylglucosaminidase and functional autolysin in *Lactobacillus acidophilus* NCFM. Appl. Environ. Microbiol..

[18] Palomino MM (2016). Influence of osmotic stress on the profile and gene expression of surface layer proteins in *Lactobacillus acidophilus* ATCC 4356. Appl. Microbiol. Biotechnol..

[19] Goh YJ (2009). Development and application of a upp-based counterselective gene replacement system for the study of the S-layer protein SlpX of *Lactobacillus acidophilus* NCFM. Appl. Environ. Microbiol..

[20] Johnson BR (2016). Conserved S-layer-associated proteins revealed by exoproteomic survey of S-layer-forming Lactobacilli. Appl. Environ. Microbiol..

[21] Simón-Soro Á (2015). Revealing microbial recognition by specific antibodies. BMC Microbiol..

[22] Chen Z, Chen L, Zhang W (2017). Tools for genomic and transcriptomic analysis of microbes at single-cell level. Front. Microbiol..

[23] Bock C, Farlik M, Sheffield NC (2016). Multi-omics of single cells: Strategies and applications. Trends Biotechnol..

[24] Cross KL (2019). Targeted isolation and cultivation of uncultivated bacteria by reverse genomics. Nat. Biotechnol..

[25] Marcos-Fernández R, Ruiz L, Blanco-Míguez A, Margolles A, Sánchez B (2019). Cell wall hydrolase as a surface-associated protein target for the specific detection of *Lactobacillus rhamnosus* using flow cytometry. Innov. Food Sci. Emerg. Technol..

[26] Sánchez B, Saad N, Schmitter JM, Bressollier P, Urdaci MC (2010). Adhesive properties, extracellular protein production, and metabolism in the *Lactobacillus rhamnosus* GG strain when grown in the presence of mucin. J. Microbiol. Biotechnol..

[27] Marjanovič I, Kandušer M, Miklavčič D, Keber MM, Pavlin M (2014). Comparison of flow cytometry, fluorescence microscopy and spectrofluorometry for analysis of gene electrotransfer efficiency. J. Membr. Biol..

[28] Wang B (2016). Altered fecal microbiota correlates with liver biochemistry in nonobese patients with non-alcoholic fatty liver disease. Sci. Rep..

[29] Castaner O (2018). The gut microbiome profile in obesity: A systematic review. Int. J. Endocrinol..

[30] Theriot CM, Young VB (2015). Interactions between the gastrointestinal microbiome and *Clostridium difficile*. Annu. Rev. Microbiol..

[31] Angelakis E, Raoult D (2018). Gut microbiota modifications and weight gain in early life. Hum. Microbiome J..

[32] Zhu W (2018). Precision editing of the gut microbiota ameliorates colitis. Nature.

[33] Hsu BB (2019). Dynamic modulation of the gut microbiota and metabolome by bacteriophages in a mouse model. Cell Host Microbe.

[34] Kant R, Paulin L, Alatalo E, de Vos WM, Palva A (2011). Genome Sequence of *Lactobacillus amylovorus* GRL1118, isolated from pig ileum. J. Bacteriol..

[35] Espino E (2015). Uncovering surface-exposed antigens of *Lactobacillus rhamnosus* by cell shaving proteomics and two-dimensional immunoblotting. J. Proteome Res..

[36] Ronda C, Chen SP, Cabral V, Yaung SJ, Wang HH (2019). Metagenomic engineering of the mammalian gut microbiome in situ. Nat. Methods.

[37] Routy B (2018). Gut microbiome influences efficacy of PD-1-based immunotherapy against epithelial tumors. Science.

[38] Gopalakrishnan V (2018). Gut microbiome modulates response to anti-PD-1 immunotherapy in melanoma patients. Science.

[39] Wirbel J (2019). Meta-analysis of fecal metagenomes reveals global microbial signatures that are specific for colorectal cancer. Nat. Med..

[40] Thomas AM (2019). Metagenomic analysis of colorectal cancer datasets identifies cross-cohort microbial diagnostic signatures and a link with choline degradation. Nat. Med..

[41] Hemarajata P, Versalovic J (2013). Effects of probiotics on gut microbiota: Mechanisms of intestinal immunomodulation and neuromodulation. Ther. Adv. Gastroenterol..

[42] Hevia A, Delgado S, Margolles A, Sanchez B (2015). Application of density gradient for the isolation of the fecal microbial stool component and the potential use thereof. Sci. Rep..

[43] Sánchez, B., Ruiz, L., Suárez, A., De los Reyes-Gavilán, C. G. & Margolles, A. Human cecum content modulates production of extracellular proteins by food and probiotic bacteria. *FEMS Microbiol. Lett.* **324**, 189–194 (2011). 10.1111/j.1574-6968.2011.02408.x 22092821

[44] Laemmli UK (1970). Cleavage of structural proteins during the assembly of the head of bacteriophage T4. Nature.

[45] Hevia A (2014). Association of levels of antibodies from patients with inflammatory bowel disease with extracellular proteins of food and probiotic bacteria. Biomed. Res. Int..

[46] Sánchez B, Schmitter J-M, Urdaci MC (2009). Identification of novel proteins secreted by *Lactobacillus rhamnosus* GG grown in de Mann-Rogosa-Sharpe broth. Lett. Appl. Microbiol..

[47] Milani C (2013). Assessing the fecal microbiota: An optimized ion torrent 16S rRNA gene-based analysis protocol. PLoS ONE.

[48] Caporaso JG (2010). QIIME allows analysis of high-throughput community sequencing data. Nat. Methods.

[49] Callahan BJ (2016). DADA2: High-resolution sample inference from Illumina amplicon data. Nat. Methods.

[50] Bokulich NA (2018). Optimizing taxonomic classification of marker-gene amplicon sequences with QIIME 2’s q2-feature-classifier plugin. Microbiome.

[51] Quast C (2012). The SILVA ribosomal RNA gene database project: Improved data processing and web-based tools. Nucleic Acids Res..

