# Supplementary material for: Precision modification of the human gut microbiota targeting surface-associated proteins
Source: Sci Rep. 2021 Jan 14;11:1270. doi: 10.1038/s41598-020-80187-3 (PMC7809461; doi:10.1038/s41598-020-80187-3)
Supplement: Supplementary file 1 — Supplementary Figures. [file 41598_2020_80187_MOESM1_ESM.pdf]

Precision modification of the human gut microbiota targeting surface-associated proteins

Raquel Marcos-Fernández[1,2], Lorena Ruiz[1,2], Aitor Blanco-Míguez[1,2], *Abelardo Margolles[1,2], *Borja Sánchez[1,2]

[1]*Department of Microbiology and Biochemistry of Dairy Products, Instituto de Productos Lácteos de Asturias – Consejo Superior de Investigaciones Científicas (IPLA-CSIC), Villaviciosa, Asturias, Spain;* [2]*Functionality and Ecology of Beneficial Microbes (MicroHealth) Group, Instituto de Investigación Sanitaria del Principado de Asturias (ISPA), Oviedo, Asturias, Spain*

* Corresponding authors:

Borja Sánchez and Abelardo Margolles, Departamento de Microbiología y Bioquímica, Instituto de Productos Lácteos de Asturias-Consejo Superior de Investigaciones Científicas (IPLA-CSIC), Paseo Río Linares s/n, 33300-Villaviciosa, Spain. Tel.: +34 985 89 21 31, Fax: +34 985 89 22 33, e-mail addresses: borja.sanchez@csic.es; amargolles@ipla.csic.es

Supplementary information

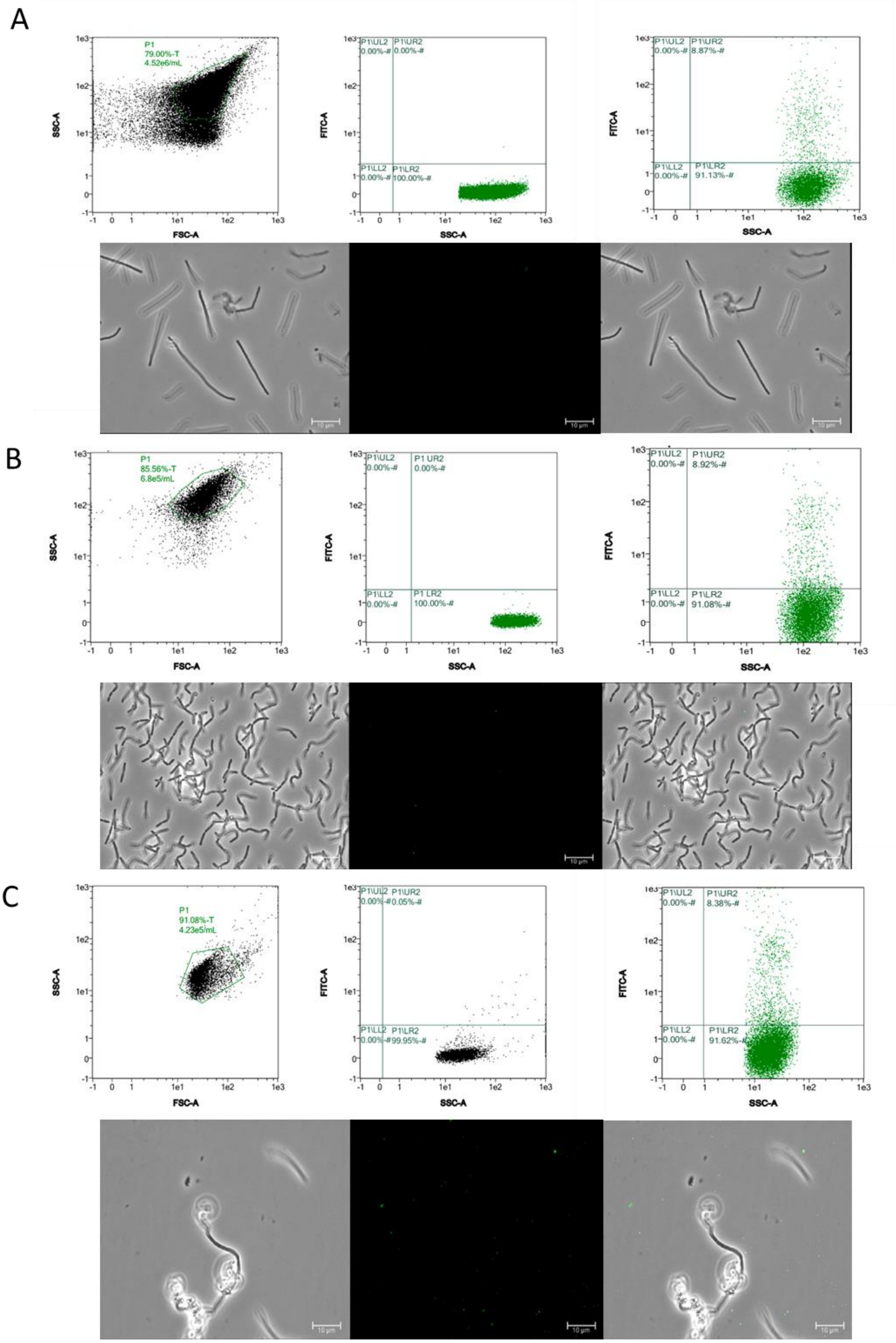
A
B
C
P1
79.00%-T
4.52e6/mL
85.56%-T
6.8e5/mL
91.08%-T
4.23e5/mL
SSC-A
FSC-A
FITC-A
P1\UL2 0.00%-#
P1\UR2 0.00%-#
P1\LL2 0.00%-#
P1\LR2 100.00%-#
P1\UR2 8.87%-#
P1\LR2 91.13%-#
P1\UR2 8.92%-#
P1\LR2 91.08%-#
P1\UR2 0.05%-#
P1\LR2 99.95%-#
P1\UR2 8.38%-#
P1\LR2 91.62%-#
10 µm

D

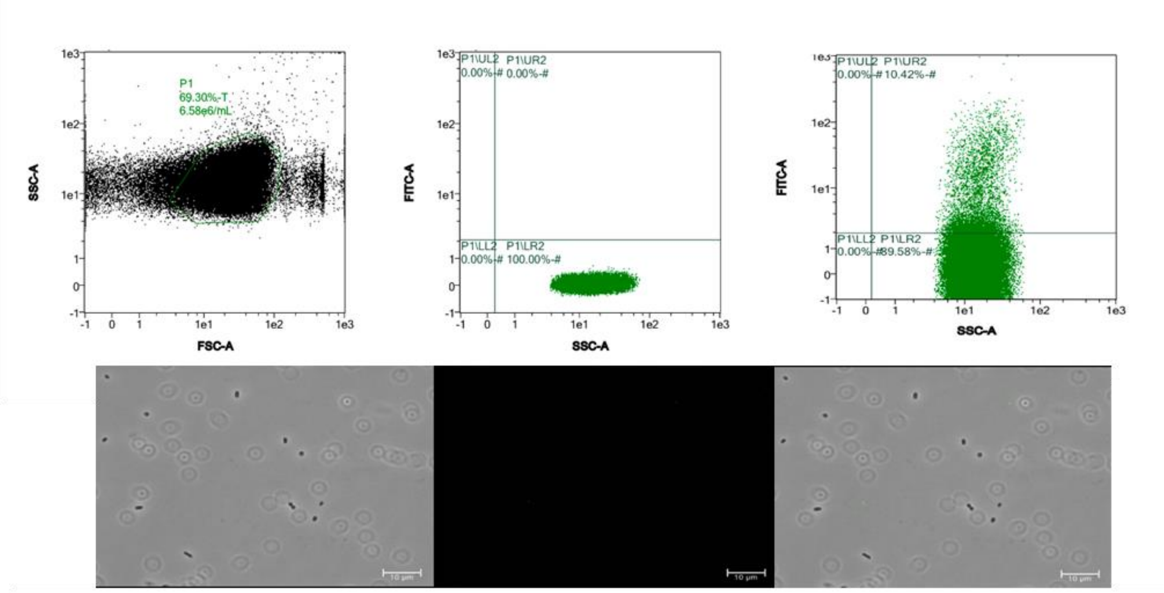
P1
69.30%-T
6.58e6/mL
SSC-A
FSC-A
FITC-A
P1\UL2 0.00%-#
P1\UR2 0.00%-#
P1\LL2 0.00%-#
P1\LR2 100.00%-#
P1\UL2 0.00%-#
P1\UR2 10.42%-#
P1\LL2 0.00%-#
P1\LR2 89.58%-#

E

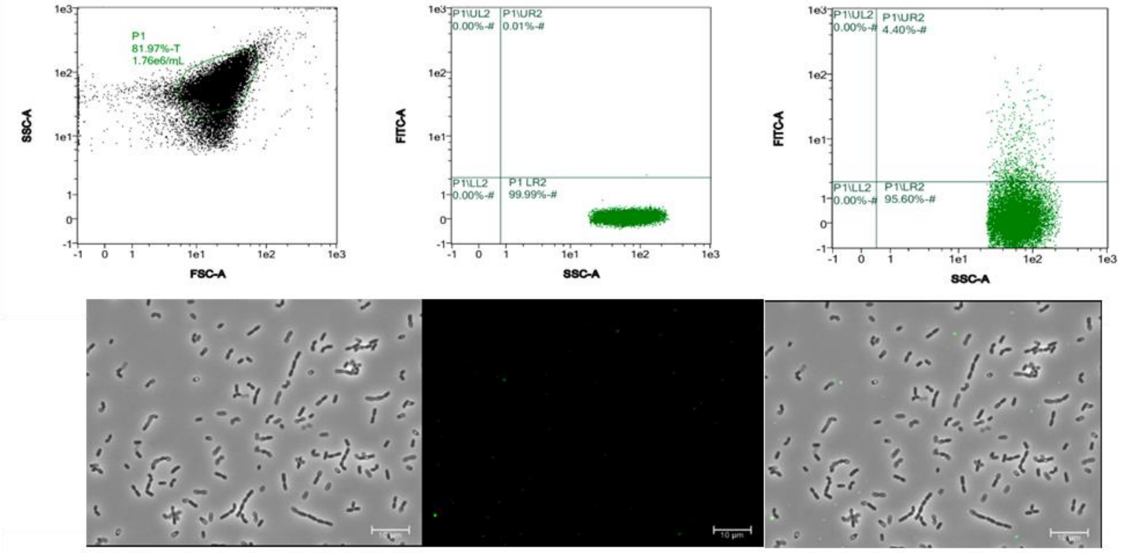
P1
81.97%-T
1.76e6/mL
SSC-A
FSC-A
FITC-A
P1\UL2 0.00%-#
P1\UR2 0.01%-#
P1\LL2 0.00%-#
P1 LR2 99.99%-#
P1\UL2 0.00%-#
P1\UR2 4.40%-#
P1\LL2 0.00%-#
P1\LR2 95.60%-#
10 µm

F

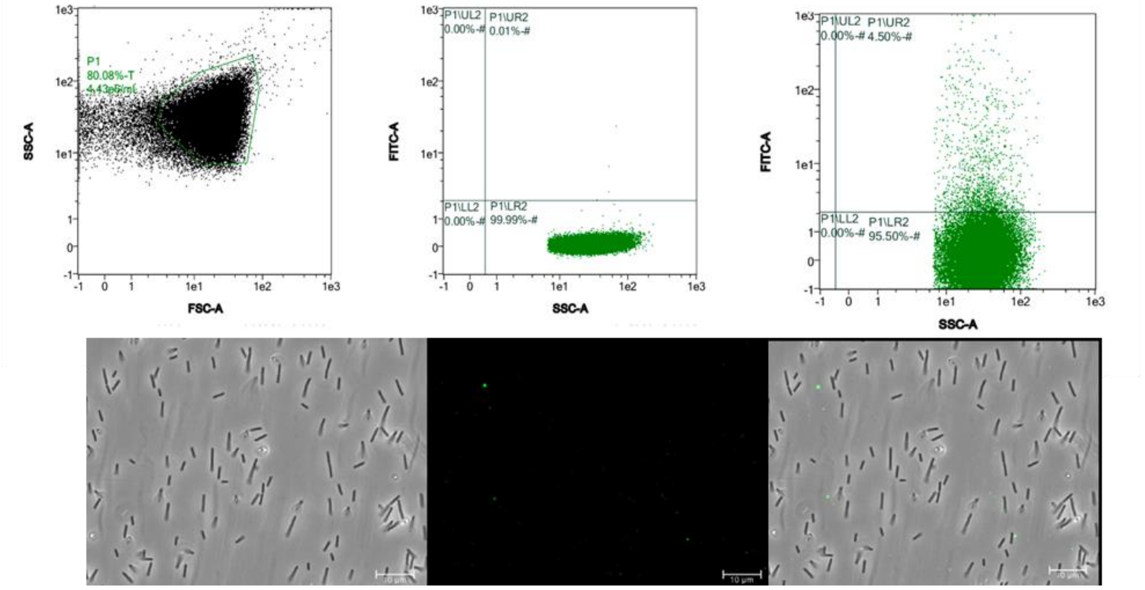
P1
80.08%-T
SSC-A
FSC-A
FITC-A
P1\UL2 0.00%-#
P1\UR2 0.01%-#
P1\LL2 0.00%-#
P1\LR2 99.99%-#
P1\UL2 0.00%-#
P1\UR2 4.50%-#
P1\LL2 0.00%-#
P1\LR2 95.50%-#
10 µm

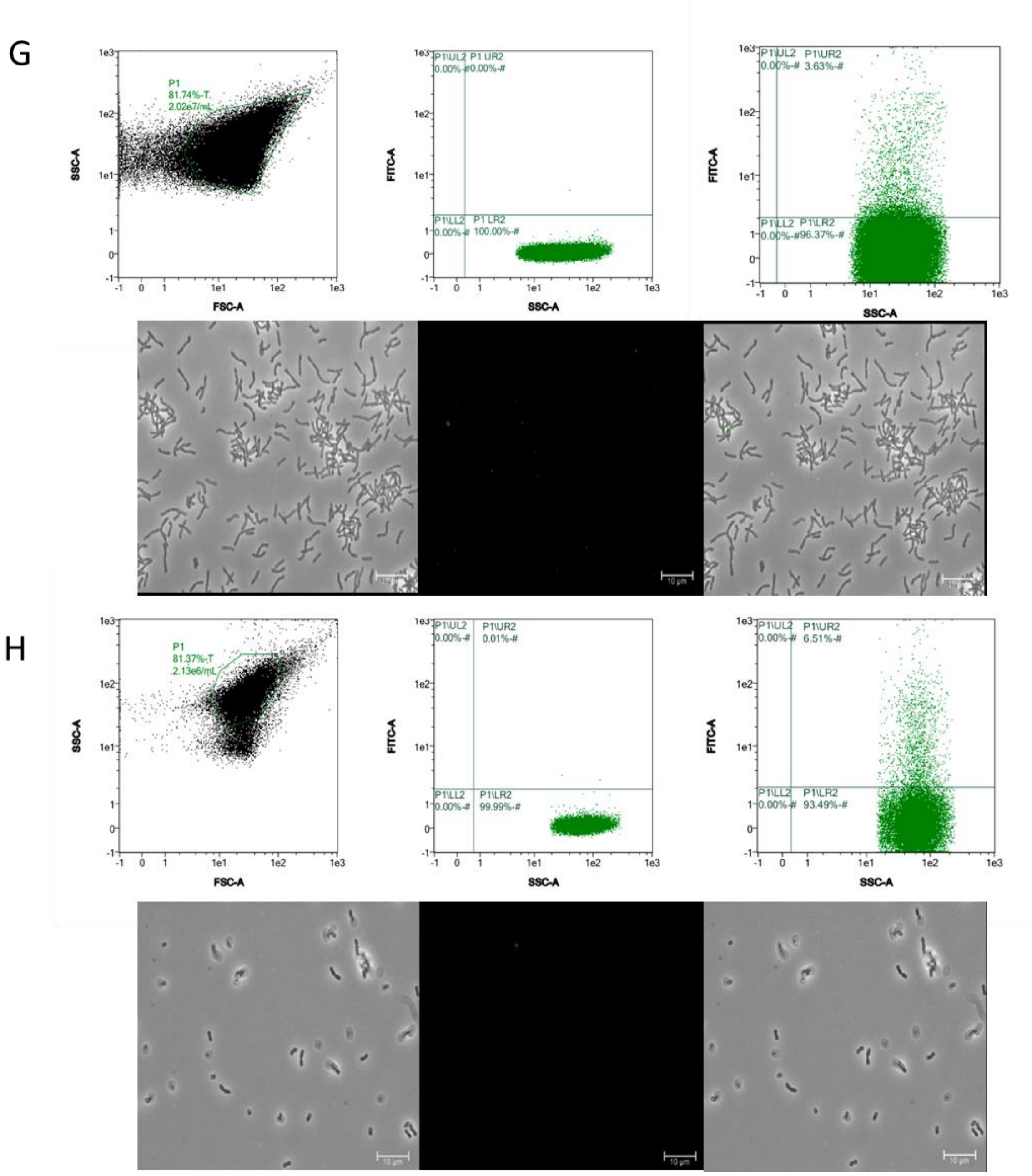

Supplementary Figure 1. Immunofluorescence microscopy data and dispersion diagrams of representative flow cytometry experiments showing the acquisition of marked and non-marked *L. amylovorus* B13 (A) *L. casei* 393 (B), *L. delbrueckii ssp. delbrueckii* IPLAlb101 (C), *E. coli* LMG2092 (D), *L. plantarum* NCIMB 8826 (E), *L. gasseri* BM7/10 (F), *B. longum* biotype *longum* NCIMB 8809 (G) and *L. reuteri* DSM20016^T (H) cell suspensions in exponential phase of growth. The labelled and unlabelled individual bacteria were assessed by triplicate.

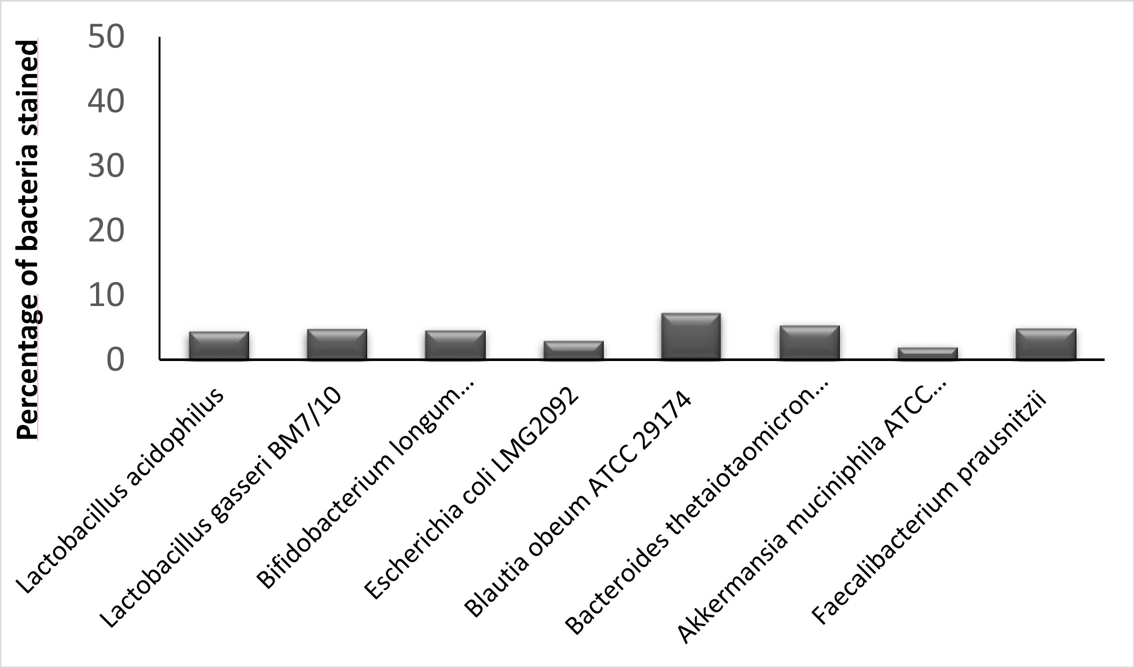

Supplementary Figure 2. Bar chart showing the percentages of bacteria labelled with preimmune serum in eight different bacterial strains.

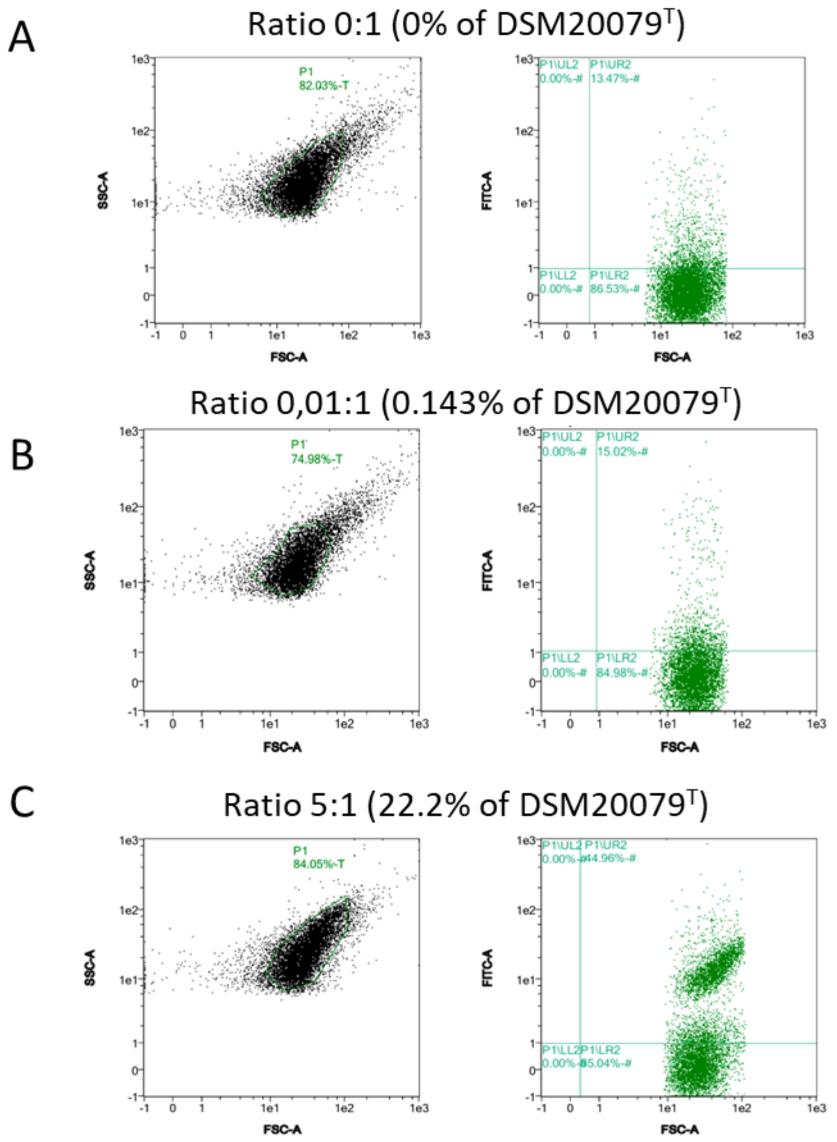

Supplementary Figure 3. Dispersion diagrams of a representative flow cytometry experiment showing the detection of *L. acidophilus* DSM20079^T in a defined bacteria consortium when added in different proportions *L. acidophilus*: any of the other bacteria. 0:1 (A), 0.01:1 (B); and 5:1 (C). The detection of DSM20079^T in a defined bacteria consortium were assessed by triplicate.

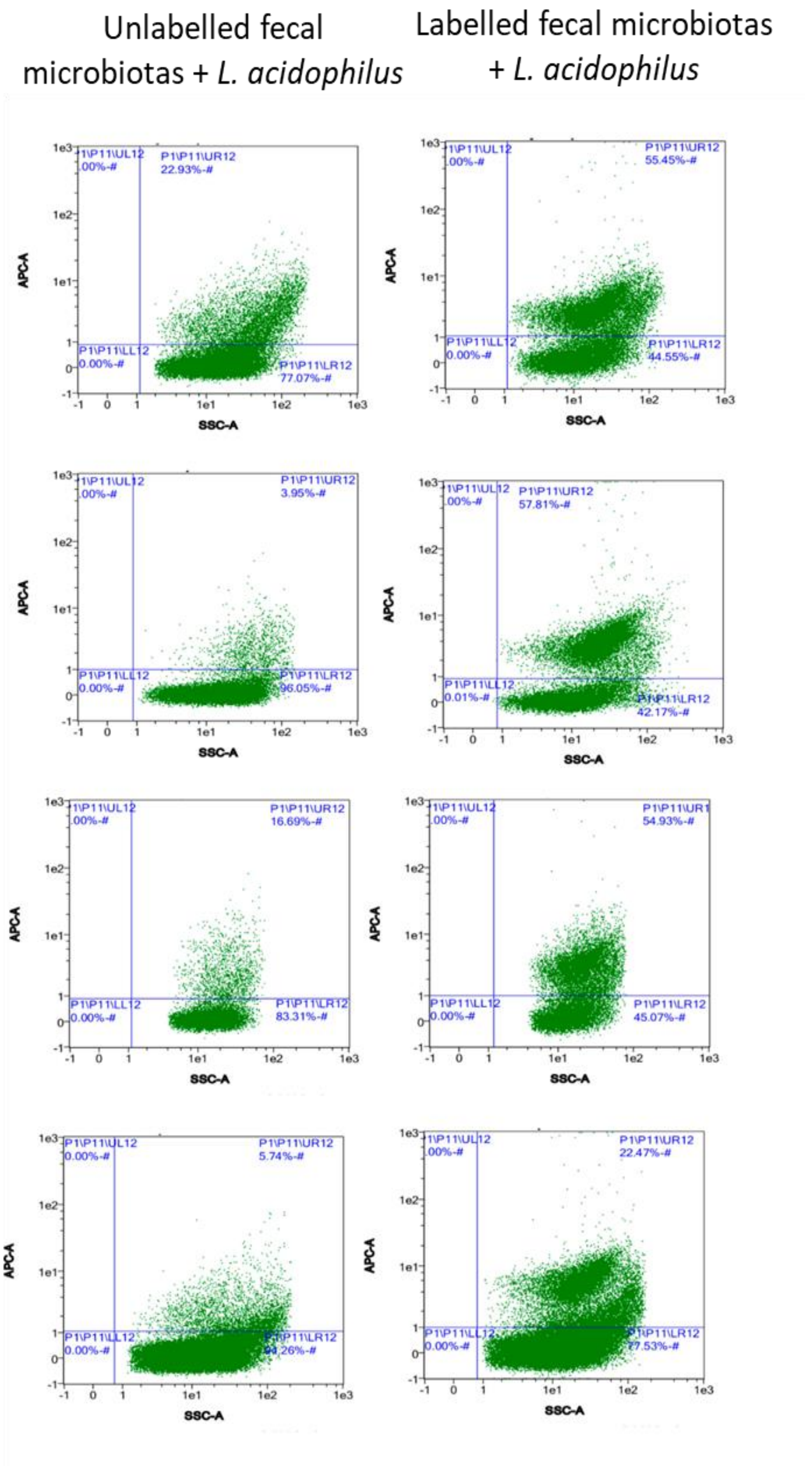

Supplementary Figure 4. Dispersion diagrams of flow cytometry experiments (a representative diagram is shown in Figure 6) showing the acquisition of unlabelled and labelled fecal microbiota supplemented with *L. acidophilus* DSM20079^T.

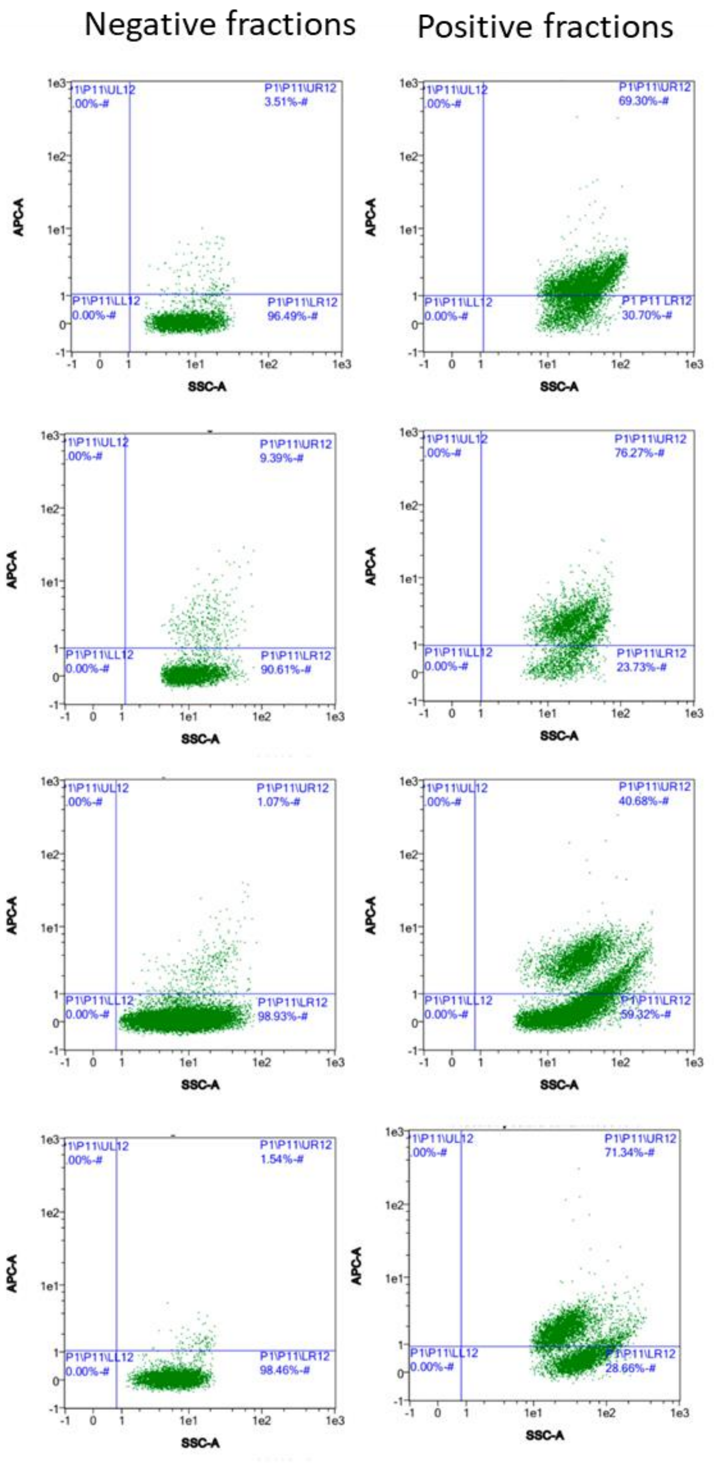

Supplementary Figure 5. Dispersion diagrams of flow cytometry experiments (a representative diagram is shown in Figure 6) showing the APC-specific fluorescence in the positive and negative fractions of a microbiota supplemented with *L. acidophilus* DSM20079^{T}

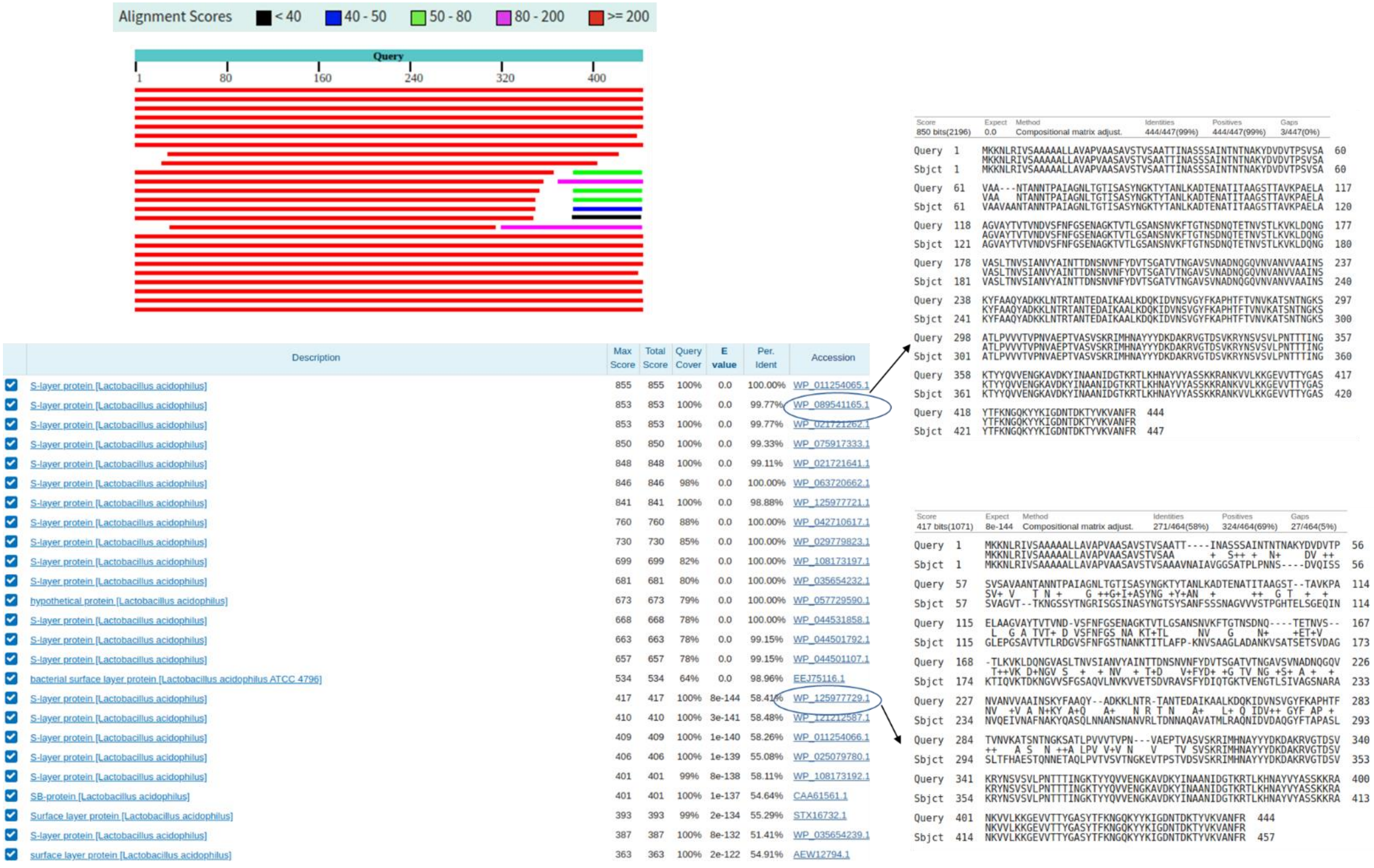

Supplementary Figure 6. Unique differences found between the SlpA protein of the *L. acidophilus* DSM20079^{T} and the public available proteomes of the *L. acidophilus* species.

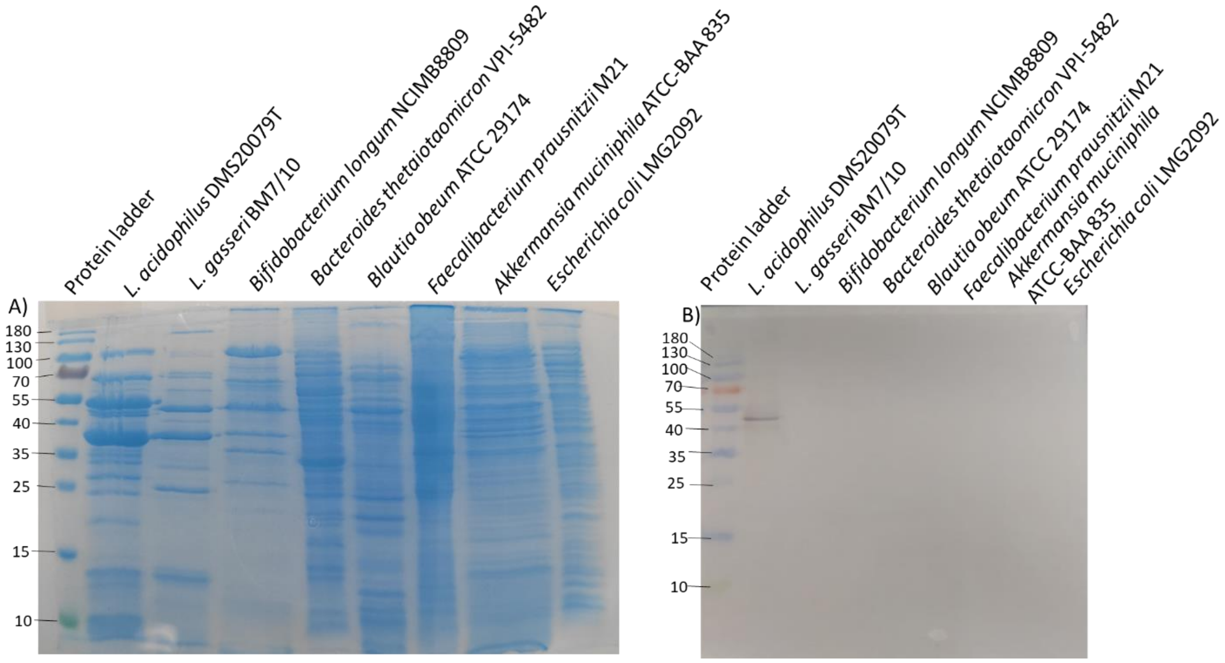

Supplementary Figure 7. (A) Representative SDS-PAGE gel showing the extracellular protein of *Lactobacillus acidophilus* DSM20079^{T}, *Lactobacillus gasseri* BM7/10, *Bifidobacterium longum* biotype *longum* NCIMB8809, *Bacteroides thetaiotaomicron* VPI-5482, *Blautia obeum* ATCC 29174, *Faecalibacterium prausnitzii* M21, *Akkermansia muciniphila* ATCC BAA-835 and *Escherichia coli* LMG2092. (B) Immunoreactive bands corresponding to SlpA of *L. acidophilus* DSM20079T labeled with the polyclonal antibody. A secondary anti-rabbit and anti-mouse IgA antibody conjugated to HRP was used.

A)

Taxonomy	F. neg 1	F. pos 1	F. neg 2	F. pos 2	F. neg 3	F. pos 3	F. neg 4	F. pos 4	F.neg 5	F. pos 5
Lactobacillus acidophilus	0.07%	2.97%	0.15%	18.78%	0.03%	28.93%	0.05%	42.70%	0.13%	21.21%

B)

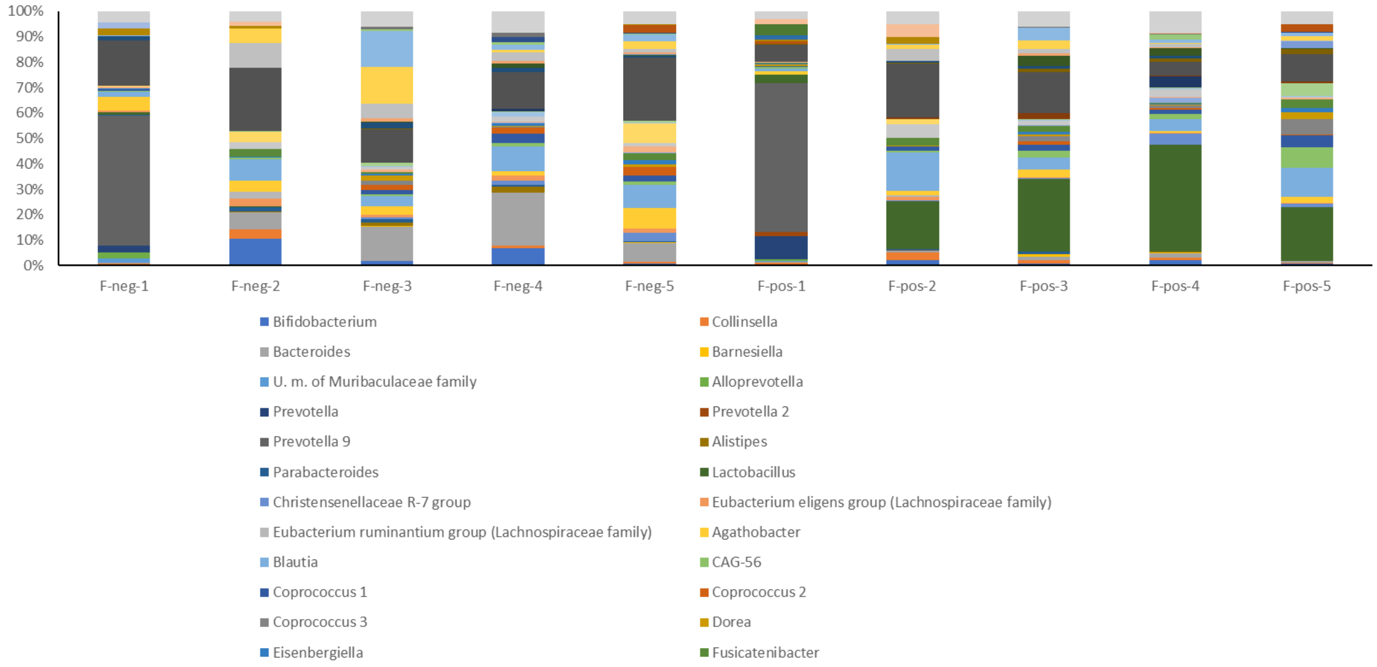

Supplementary Figure 8. (A) Relative abundance of *Lactobacillus* in depleted (negative) and enriched (positive) fractions of five microbiotas supplemented with *L. acidophilu.s* (B) Genera present in positive and negative fractions of the microbiotas supplemented with *L. acidophilus.*
